# Deep brain stimulation for psychiatric disorders: role of imaging in identifying/confirming DBS targets, predicting, and optimizing outcome and unravelling mechanisms of action

**DOI:** 10.1093/psyrad/kkab012

**Published:** 2021-10-04

**Authors:** Dejan Georgiev, Harith Akram, Marjan Jahanshahi

**Affiliations:** Department of Clinical and Movement Neurosciences, UCL Queen Square Institute of Neurology, Queen Square, London, WC1N 3BG, UK; Department of Neurology, University Medical Centre Ljubljana, Zaloška cesta 2, 1000 Ljubljana, Slovenia; Artificial Intelligence Laboratory, Faculty of Computer and Information Science, University of Ljubljana, Večna pot 113, 1000 Ljubljana, Slovenia; Department of Clinical and Movement Neurosciences, UCL Queen Square Institute of Neurology, Queen Square, London, WC1N 3BG, UK; Department of Clinical and Movement Neurosciences, UCL Queen Square Institute of Neurology, Queen Square, London, WC1N 3BG, UK; The Clinical Hospital of Chengdu Brain Science Institute, MOE Key Laboratory for Neuroinformation, University of Electronic Science and Technology of China, Chengdu, 611731, China

**Keywords:** deep brain stimulation, psychiatric disorders, major depressive disorder, obsessive–compulsive disorder, dementia

## Abstract

Following the established application of deep brain stimulation (DBS) in the treatment of movement disorders, new non-neurological indications have emerged, such as for obsessive–compulsive disorders, major depressive disorder, dementia, Gilles de la Tourette Syndrome, anorexia nervosa, and addictions. As DBS is a network modulation surgical treatment, the development of DBS for both neurological and psychiatric disorders has been partly driven by advances in neuroimaging, which has helped explain the brain networks implicated. Advances in magnetic resonance imaging connectivity and electrophysiology have led to the development of the concept of modulating widely distributed, complex brain networks. Moreover, the increasing number of targets for treating psychiatric disorders have indicated that there may be a convergence of the effect of stimulating different targets for the same disorder, and the effect of stimulating the same target for different disorders. The aim of this paper is to review the imaging studies of DBS for psychiatric disorders. Imaging, and particularly connectivity analysis, offers exceptional opportunities to better understand and even predict the clinical outcomes of DBS, especially where there is a lack of objective biomarkers that are essential to properly guide DBS pre- and post-operatively. In future, imaging might also prove useful to individualize DBS treatment. Finally, one of the most important aspects of imaging in DBS is that it allows us to better understand the brain through observing the changes of the functional connectome under neuromodulation, which may in turn help explain the mechanisms of action of DBS that remain elusive.

## Introduction

The burden of psychiatric disorders is immense (Wittchen *et al*., 2011) and continues to increase. Many of the patients suffering from these disorders can be treated with the usual, non-invasive methods, including medication and/or psychotherapy. However, there is a group of patients for whom the conventional therapies are not effective. Indeed, psychosurgery was developed because of the need to manage untreatable psychiatric disorders, starting with Edgar Moniz’s prefrontal leucotomy to section the connections between the prefrontal cortex (PFC) and the thalamus (Faria, 2013). This was later followed by the infamous Freeman and Watts’ “refinement” of PFC leucotomy by the use of the transorbital approach (Freeman and Watts, 1942). Since then, the number of procedures to treat psychiatric disorders rapidly grew, reaching its peak in the 1950s, and included anterior capsulotomy (Christmas *et al*., 2011), subcaudate tractotomy (Hodgkiss *et al*., 1995), anterior cingulotomy (Shields *et al*., 2008), and limbic leucotomy (Cho *et al*., 2008) for major depressive disorder (MDD) and anterior capsulotomy for obsessive–compulsive disorder (OCD) (Pepper *et al*., 2019). A decline in the use of psychosurgery to treat psychiatric disorders followed, mainly due to the advent of psychopharmacology (Vetrano *et al*., 2021). However, over recent years, due to the development of neuroscience and neurosurgical technology, and the success of deep brain stimulation (DBS) for movement disorders, there has been a revival of neurosurgical procedures for the treatment of psychiatric disorders (Vetrano *et al*., 2021), albeit mainly for OCD and MDD (Graat *et al*., 2017).

DBS, which now has an important and well-established role in the treatment of movement disorders, involves the insertion of electrodes into specific brain targets and delivery of electrical current to those regions by means of an implanted pulse generator in the chest area. Following the great success of DBS in treating movement disorders, as well the positive effect of DBS in OCD (Rauch *et al*., 2006; Baldermann *et al*., 2019a; Tyagi *et al*., 2019; Li *et al*., 2021) and Gilles de la Tourette Syndrome (GTS) (Ackermans *et al*., 2011; Kefalopoulou *et al*., 2015a), new indications for DBS have emerged, such as MDD (Schlaepfer *et al*., 2008; Riva-Posse *et al*., 2018), anorexia nervosa (Israel *et al*., 2010), aggressive behaviour (Sano and Mayanagi, 1988; Torres *et al*., 2020), post-traumatic stress disorder (PTSD) (Langevin *et al*., 2016), dementia (Turnbull *et al*., 1985; Laxton *et al*., 2010; Kuhn *et al*., 2015a; Kuhn *et al*., 2015b; Gratwicke *et al*., 2018; Gratwicke *et al*., 2020), and addiction (Muller *et al*., 2009). Except for OCD and GTS, most of these applications, remain experimental. The development of DBS for both neurological and psychiatric disorders has been driven primarily by advances in neuroimaging and electrophysiology. Another factor leading to the increasing use of DBS in neurology and psychiatry is its minimally invasive nature, combined with the low incidence of severe side effects. This has led to new strategies for better targeting, identification of optimal stimulation sites, and a better understanding of the mechanisms of action of DBS in various diseases.

Even though the concept of modulating of white matter pathways is not new and dates back to the 1950s, when Talairach and Leksell introduced anterior capsulotomy (Ballantine, 1988; Feldman and Goodrich, 2001), only recently has DBS undergone a conceptual paradigm shift away from stimulation of a specific brain nucleus to modulation of widely distributed, complex brain networks (Horn and Fox, 2020; Sullivan *et al*., 2020; Wang *et al*., 2021). This was made possible by the fact that recent developments in magnetic resonance imaging (MRI) technology, such as diffusion-weighted imaging-based tractography, are increasingly being used to more accurately target brain regions thought to be important in the generation of psychiatric disorders (Coenen *et al*., 2017). Imaging has been used also in DBS for movement disorders in a variety of aims: pre-surgical screening, target selection, neurosurgical planning, post-surgical confirmation of electrode position, evaluating treatment effects, and examination of clinical correlations (Peng *et al*., 2021). Moreover, the increasing number of targets for treating psychiatric disorders indicates that there may be a convergence of the effect of stimulating different targets for the same disorder and also the effect of stimulating the same target for different disorders (Horn and Fox, 2020; Sullivan *et al*., 2020). Nevertheless, there is still a lack of knowledge regarding the optimal DBS targets for psychiatric disorders. Moreover, even less is known about the exact mechanisms of action of DBS for these disorders, which probably involves not only modulation of brain circuits (Sullivan *et al*., 2020), but also induction of a range of cellular, molecular, and neuroplastic changes in the brain (Jakobs *et al*., 2019). Thus, the search for optimal targeting and understating of the mechanism of action of DBS for various psychiatric disorders is still ongoing.

The main aim of this article is to review the literature on the use of imaging in identifying DBS targets and determining the mechanisms of action of DBS in psychiatric disorders. We will first present the studies on OCD, then on MDD, GTS, dementia, anorexia nervosa, and finish with a review of the relevant literature on addiction, PTSD, and schizophrenia, before discussing the implications for treatment of these disorders with DBS.

## Materials and Methods

A systematic literature search for publications on DBS in psychiatric disorders was conducted on the PUBMED database in November 2020. The search algorithm included DBS, imaging, connectivity, functional (fMRI), positron-emission tomography-computed tomography (PET-CT), and psychiatric disorders, depression, OCD, GTS, dementia, anorexia nervosa, and PTSD. Reference lists of relevant studies were also reviewed. Studies on humans were limited to English language. No restrictions were placed on study design or the number of participants. A total of 161 articles were identified and reviewed in full text. After this elimination, a total of 57 studies were considered for this review. The imaging studies included in this review are listed in Tables 1–3. The identified studies were analysed based on indication, DBS target areas, patient characteristics, key outcome measures, imaging modality used, and treatment results.

**Table 1:** Studies of DBS for OCD using imaging.

	Study	Study design	Target	*N*	Outcome measure	Imaging modality/connectivity type/connectivity data	Results
1	Rauch 2006	Prospective	VC/VS	6 OCD	Y-BOCS	FDG-PET	Acute DBS of the VC/VS was associated with activation of the fronto-thalamo-basal ganglia-thalamic circuit including activation of OFC, ACC, striatum, GP, and thalamus.
2	Figee 2013	Retrospective, quasi experimental	NAcc	16 OCD/13 HC	Y-BOCS	MRI/functional/individualized	Resting-state fMRI scans revealed that DBS reduced the connectivity between the NAcc and the lateral PFC and medial PFC.
3	Suetens 2014	Retrospective	BNST	13 capsulotomy/16 BNST-DBS OCD	Y-BOCS	FDG-PET	Capsulotomy and DBS lead to similar clinical improvement and similar metabolic network changes in the CSTC circuit, especially in the subgenual anterior cingulate, but the changes metabolic changes were more pronounced and extended in capsulotomy.
4	Hartmann 2015	Retrospective	ALIC/NAcc	6 OCD	Y-BOCS	MRI/structural/normative	Modulation of the right DLPFC was associated with good DBS response, activation of the right lOFC was associated with poor DBS response.
5	Dougherty 2016	Prospective, uncontrolled	VC/VS	6 OCD	Y-BOCS, HAM-D	FDG-PET	Perfusion in the dACC significantly increased when monopolar DBS was turned on at the most ventral DBS contact, and this was correlated with reductions in depressive symptom severity.
6	Makris 2016	Retrospective, quasi-experimental	VC/VS or ALIC/NAcc	1 OCD/29 HC	Y-BOCS	MRI/structural/normative and individualized	The active contact at both electrodes were located within the tracts related to lateral OFC and medial OFC. A patient-specific approach, using DTI can enhance the efficacy of DBS for OCD.
7	Coenen 2017	Case reports	sIMBF	2 OCD	Y-BOCS	MRI/structural/individualized	DTI used to precisely implant the target in two patients with good effect of stimulation.
8	Voon 2017	Retrospective, quasi-experimental	STN	12 OCD/24 HC 154 HC (rs-fMRI)	Y-BOCS, Beads task to test for reflective impulsivity	MRI/functional/individualized	Patients with OCD with STN-DBS may be less likely to accumulate evidence (i.e. they make more impulsive choices) with no difference in subjective confidence on vs. off stim. Stimulation of the associative and limbic STN is associated with increased impulsivity in OCD patients on stimulation.
9	Balderman 2019	Prospective	VC/VS	3 OCD	Y-BOCS	FDG-PET	VC/VS-DBS for OCD increases brain metabolism locally. Across distributed global networks, DBS appears to exert differential effects, possibly depending on localization of stimulation sites and response to the intervention.
10	Balderman 2019	Prospective	ALIC/NAcc	22 OCD	Y-BOCS	MRI/structural/individualized and normative	The degree of connectivity between stimulation sites and medial and lateral PFC significantly predicted clinical improvement. A fronto-thalamic pathway that is crucial for beneficial outcome was also identified.
11	Balderman 2019–1	Retrospective	VC/VS	25 OCD	Y-BOCS, weight	MRI/functional/normative	Increase of weight after DBS, associated with medial/apical localization of stimulation sites and with connectivity to hypothalamic areas and the BNST.
12	Barcia 2019	Prospective	NAcc/VS	7 OCD	Y-BOCS	MRI/structural and functional/individualized	6 patients were classified as responders, with median symptomatic reduction of 50% achieved from each patient's best contact. This was located at the caudate in 4 cases and at the NAcc in 2.
13	Lee 2019	Prospective	ITP	5 OCD	Y-BOCS	FDG-PET	All 5 patients were considered responders at one year and last follow-up. Post-operative FDG-PET imaging in 2 patients demonstrated decreased glucose uptake within the right caudate, right putamen, right SMA, and right cingulum and increased glucose uptake in bilateral motor areas, left temporal pole, and left OFC.
14	Liebrand 2019	Retrospective	ALIC	12 OCD	Y-BOCS	MRI/structural/individualized	Active stimulation of the vALIC closer to the MFB than the ATR was associated with better treatment outcome.
15	Park 2019	Prospective	ALIC/NAcc	2 ALIC-DBS/2 NAcc-DBS	Y-BOCS	FDG-PET	DBS was effective no matter the target. FDG-PET imaging indicated post-surgical reductions in metabolism, in not only targeted limbic networks, but also frontal cortical and subcortical regions, suggesting that large-scale network modulation and inhibition are associated with functional recovery in OCD.
16	Tyagi 2019	Randomized Controlled Trial	amSTN and VS/VS	6 OCD	Y-BOCS	MRI/structural/individualized	amSTN significantly improved cognitive flexibility, whereas VC/VS-DBS had a greater effect on mood. VC/VS-DBS connected primarily to the mOFC, and amSTN DBS to the lOFC, and DLPFC.
17	Fridgeirsson 2020	Prospective	vALIC	16 OCD	Y-BOCS	MRI/functional/individualized	Improvement in mood and anxiety following vALIC-DBS was associated with reduced amygdala-insula functional connectivity. Directional (effective) connectivity analysis revealed that DBS increased the impact of the ventromedial PFC on the amygdala and decreased the impact of the amygdala on the insula.
18	Li 2020	Multicentric retrospective	ALIC/NAcc and STN	50 OCD	Y-BOCS	MRI/structural/normative	A bundle connecting frontal lobe to STN was associated with optimal clinical response targeting either ALIC, NAcc or STN. When informing the tract target based on the first cohort, clinical improvements in the second could be significantly predicted, and vice versa.
19	Mosley 2021	Randomized, double blind, sham controlled	BNST/NAcc	9 OCD	Y-BOCS	MRI/structural/normative	In the blinded phase, there was a significant benefit of active stimulation over sham. After the open phase, 7 participants classified as responders. Structural connectivity revealed isolated right-hemispheric fibres associated with OCD imporvement.
20	Liebrand 2021	Retrospective	NAcc	57 OCD	Y-BOCS	Structural MRI	Patients with larger NAcc volumes show a better response to DBS, indicating that DBS success is partly determined by individual differences in brain anatomy. Structural MRI data alone does not provide sufficient information to guide clinical decision making at an individual level.
21	Li 2021	Multicentric retrospective	ALIC/NAcc and STN	50 OCD	Y-BOCS	MRI/functional/normative	Connectivity to ACC, insula, and precuneus, among other regions, was predictive regardless of stimulation target. Regions with maximal connectivity to these commonly predictive areas included insula, superior frontal gyrus, ACC, and anterior thalamus, as well as the original stereotactic targets.

DTI = diffusion tensor imaging, FDG-PET = fluorodeoxy glucose-PET, HAM-D = Hamilton Depression Rating Scale, HC = healthy control participants.

**Table 2:** Studies of DBS in MDD using imaging.

	Study	Study design	Target	*N*	Outcome measure	Imaging modality/connectivity type/connectivity data	Results
1	Schlaepfer 2007	Prospective, pilot	NAcc	3	HDRS	FDG-PET	Clinical improvement in all 3 patients. FDG-PET showed significant changes in the fronto-striatal networks
2	Schlaepfer 2013	Prospective, pilot	slMFB	7	MADRS	MRI/structural/individualized	Bilateral slMFB significantly reduced symptoms in treatment-resistant MDD in 6/7 patients. Tractography was helpful in planning the target as 4/7 patients were remitters and needed lower intensities of stimulation with less side effects.
3	Riva-Posse 2014	Prospective, single blind	Cg25	17	HDRS-17	MRI/structural/individualized	At 6 months there were 7 responders, at 2 years there were 13 responders. Responders shared bilateral pathways from their activation volumes to (i) medial frontal cortex via forceps minor and uncinate fasciculus, (ii) rostral and dorsal cingulate cortex via the cingulum bundle, and (iii) subcortical nuclei.
4	Choi 2015	Experimental, intra-operative testing of acute behavioural effects	Cg25	9	Self-reports	MRI/structural/individualized	All 9 patients had a good response to Cg25-DBS intra-operatively. Structural connectivity showed that the best response contacts had a pattern of connections to the bilateral ventromedial frontal cortex and to the cingulate cortex, whereas behaviourally salient contacts had only cingulate involvement.
5	Martin-Bianco 2015	Retrospective	Cg25	7	HAM-D-17	FDG-PET	Inactive stimulation was characterized by metabolism decreases in dorsal anterior cingulate, premotor region and putamen with respect to active stimulation. No clinical changes according to HAM-D-17 were detected.
6	Accolla 2016	Prospective	Cg25	5	HDRS-24 and BDI	MRI/structural/individualized	Only one patient responded to Cg25-DBS, however, the stimulating contacts were localized in the pGR bilaterally, by the modulation of circuits involving mainly the medial PFC.
7	Bewernick 2017	Prospective	slMFB	8	MADRS	MRI/structural/individualized	Use of deterministic DTI for targeting slMFB: 6 of 8 patients responded well on stimulation after 12 months, and 4 patients had a long-term remission, indicating an acute and sustained effect of bilateral slMFB-DBS.
8	Riva-Posse 2018	Prospective	Cg25	11	HDRS-17	MRI/structural/individualized	A probabilistic tract map of all participants demonstrated inclusion of the four bundles as intended, matching the connectome blueprint previously defined: 8 of 11 patients were responders and 5 were remitters after 6 months of open-label stimulation. At one year, 9 of 11 patients were responders, with 6 of them in remission.
9	Coenen 2018	Retrospective analysis of prospective data	slMFB	24	MADRS	MRI/structural/individualized and normative	A detailed description of the surgical procedure of slMFB targeting in 24 patients form two open-label trials (FORSEE and FORSEE II) is given. The results of the first study show a good response in 6 of 7 patients.
10	Fenoy 2018	Prospective, single blinded	slMFB	6	MADRS	MRI/structural/individualized	6 patients were enrolled in the study, 3 of 6 patients at one week responded on stimulation, one patient dropped out, 4 of the 5 remaining patients had a very good response at 52-weeks post-operation. Evaluation of modulated fibre tracts revealed significant common orbitofrontal connectivity to the target region in all responders. PET scan showed no change in the metabolism pattern after the stimulation.
11	Coenen 2019	Retrospective analysis of prospective data	slMFB	24	MADRS	MRI/structural/individualized and normative	A left fronto-polar and partly orbitofrontal region was identified that showed increased volume in pre-operative anatomical scans.
12	Brown 2020	Prospective	Cg25	20	HDRS	FDG-PET	10 patients were classified as responders, 5 in the subgroup of long pulse width stimulation and 5 in the short pulse width stimulation. Baseline Cg25 metabolism was significantly higher in responders than in non-responders, and this was predictive of favourable clinical response of DBS. DBS decreased the activity of Cg25 6 months after the stimulation.

Cg25 = subcallosal cingulate gyrus, HAM-D/HDRS(-17/-24) = Hamilton Depression Rating Scale-17/24 items, pGR = posterior gyrus rectus.

**Table 3:** Studies of DBS in GTS, dementia, and anorexia nervosa using imaging.

	Study	Study design	Target	*N*	Diagnosis/primary outcome measure	Imaging modality/connectivity type/connectivity data	Results
**GTS**							
1	Haense 2016	Prospective	GPi/CM-VOI	5 + 6 HC	GTS/YGTSS	Brain perfusion using 99mTc-ECD SPECT	In patients pre-operatively, the perfusion was reduced in frontal, central and parietal regions and there was an increased perfusion in the cerebellum. Both, the stimulation of GPi and CM-VOI increased the perfusion in the frontal regions and decreased it in the cerebellum.
2	Jo 2018	Prospective	CM-Pf	5	GTS/MRVRS	MRI/functional/individual	Thalamic stimulation induced suppression of motor and insula networks correlated with motor tic reduction, while suppression of frontal and parietal networks correlated with vocal tic reduction.
3	Brito 2019	Retrospective	CM-Pf	5	GTS/YGTSS	MRI/structural/normative	No relationships were found between the areas stimulated and the changes in patient tics. However, the right frontal middle gyrus, the left frontal superior sulci region and the left cingulate sulci region structurally correlated with tic improvement.
4	Johnson 2019	Retrospective	CM, Gpi, NAcc/ALIC	110	GTS/YGTSS	MRI/only structural imaging used, no connectomic analysis.	Tics and OCB improved in all patients regardless of the target, the median time to improvement was 13 months. The active contacts were clustered near the target nuclei, there were regions within and surrounding GPi and CM thalamus that improved tics for some patients but were ineffective for others. Furthermore, regions within or superior/medial to GPi, rather than inferior to GPi, were associated with greater improvement in OCB.
5	Johnson 2020a	Retrospective	CM, GPi	66 (32 CM + 34 Gpi)	GTS/YGTSS	MRI/structural/normative	The connectivity to limbic networks, associative networks, caudate, thalamus, and cerebellum was positively correlated with improvement in tics in GPi-DBS. For CM-thalamic-DBS, connectivity to sensorimotor networks, parietal-temporal-occipital networks, putamen, and cerebellum was positively correlated with tic improvement. For OCB, both targets showed that connectivity to the PFC, OFC, and cingulate cortex was positively correlated with improvement.
6	Johnson 2020b	Retrospective	GPi	35	GTS/YGTS, Y-BOCS	MRI/structural/normative	The best-fit model of tic improvement included baseline severity and the associative pallido-subthalamic pathway. The best-fit model of OCB improvement included baseline severity and the sensorimotor pallido-subthalamic pathway, with substantial evidence also supporting the involvement of the prefrontal, motor, and premotor internal capsule pathways.
7	Morishita 2020	Prospective	CM	8	GTS/YGTSS, Y-BOCS, HAM-D	MRI/structural/normative	Fibres of therapeutic stimulation were characterized by more dense connections with the precentral gyrus than were those of side effects. Specifically, dizziness was related to fibres into the cerebello-rubral network. The paraesthesia symptoms were characterized by the fibres that connect the thalamus and insular cortex. Depression was characterized by fibres that connect the thalamus with the amygdala and the OFC.
8	Andrade 2020	Retrospective	CM-VOI	7	YGTSS	MRI/structural	The results of the study show that an increase in the density of fibre projections to the seed regions of the motor cortex defined in the study—pre-SMA, SMA, and M1 is associated with better clinical outcomes. Activation of fibre projections to pre-SMA was higher in responders. The non-responders showed more diffuse stimulation of several cortical areas simultaneously, including pre-SMA, SMA and M1. The results indicate that more selective connectivity produces better clinical outcomes.
9	Kakusa 2021	Case study	CM/VC/VS	1	YGTSS	MRI/structural/normative	Tractography revealed high connectivity from the CM and VLp to the superior frontal gyrus, rostral middle frontal gyrus, brainstem, and ventral diencephalon.
**Dementia**							
1	Turnball 1985	Case study/prospective	NBM	1	AD	FDG-PET	Single electrode was placed in the left NBM. No clinical effect of stimulation, but 9 months later, on the side of the stimulation the cortical metabolic activity was preserved, despite the fact it had declined in the other parts of the cortex.
2	Freund 2009	Case study/prospective	NBM and STN	1	PDD	MRI	Turning on the STN electrodes improved motor symptoms but left cognitive performance almost unchanged. Turning on electrical stimulation of the NBM markedly improved cognitive functions.
3	Laxton 2010	Prospective, open label	Fornix	6	AD/ADAS-Cog/MMSE	sLORETA/FDG-PET	DBS drove neural activity in the memory circuit, including the entorhinal, and hippocampal areas and activated the brain's DMN. PET scans showed an early and striking reversal of the impaired glucose use in the temporal and parietal lobes that was maintained after 12 months of continuous stimulation. There was no clear clinical effect of DBS on cognition, but the post hoc evaluation of the ADSS-cog and the MMSE suggested possible improvements and/or slowing in the rate of cognitive decline at 6 and 12 months in some patients. There were no serious adverse events.
4	Smith 2012	Prospective, open label	Fornix	5	AD	FDG-PET/functional	Additional analysis of the data from Laxton 2010. Functional connectivity analyses revealed that 1 year of DBS increased cerebral glucose metabolism in 2 orthogonal networks: a frontal-temporal-parietal-striatal-thalamic network and a frontal-temporal-parietal-occipital-hippocampal network. In similar cortical regions, higher baseline metabolism prior to DBS and increased metabolism after 1 year of DBS were correlated with better outcomes in global cognition, memory, and quality of life.
5	Fontaine 2013	Case study	Fornix	1	AD/ADAS-Cog, MMSE, FCSRT	FDG-PET	After 1 year of stimulation, the memory scores were stabilized compared to baseline, and mesial temporal lobes metabolism increased as assessed by FDG-PET.
6	Kuhn 2015a	Prospective, double-blind, randomized, phase I study	NBM	6	AD/ADAS-Cog	FDG-PET	Based on stable or improved primary outcome parameters 12 months after surgery, 4 of the 6 patients were considered responders. No severe or non-transitional side effects related to the stimulation were observed. The metabolism of the whole brain, as well as of the parietal and temporal, including the amygdalo-hippocampal region increased.
7	Kuhn 2015b	Case reports	NBM	2	AD/ADAS-Cog, MMSE	MRI	Patient 1 was stable during the first year and showed deterioration in ADAS-Cog 2 years later. Patient 2 was stable (ADAS-Cog), and MMSE even improved two years after the operation. The results indicated that NBM-DBS performed at an earlier stage of the disease and at younger age may have a favourable impact on disease progression and cognitive functions
8	Lozano 2016	Prospective, double blind, randomized	Fornix	42	AD/ADAS-Cog, CDR-SB	FDG-PET	There were no differences in cognitive outcomes for participants, but participants aged 65 years or more may have derived benefit while there was possible worsening in patients below age 65 years with stimulation.
8	Balderman 2018	Mixed design, 6 retrospective and 4 prospective	NBM	10	AD/ADAS-Cog, MMSE	MRI	A fronto-parieto-temporal pattern of cortical thickness was found to be associated with beneficial outcome. Modulation of streamlines connected to left parietal and opercular cortices was associated with better response to the intervention. The results indicate that patients with less advanced atrophy may profit from DBS of the NBM.
10	Gratwicke 2018	Prospective, double blind, crossover, randomized	NBM/Some contacts in GPi	6	PDD/Many tests (see results)	MRI/functional/normative	No improvement on the primary outcome measures—CVLT-II, WAIS-III, digit span, verbal fluency, Posner covert attention test, and simple and choice reaction times. There was, however, an improvement at a group level on the NPI total score, mainly due to improvement of visual hallucinations. No effect of stimulation on the DMN at group level. MDS-UPDRS-IV measuring dyskinesias improved after the stimulation probably due to current spread to GPi.
11	Gratwicke 2020	Prospective, double blind, crossover, randomized	NBM/Some contacts in GPi	6	LBD/many tests (see results)	MRI/functional/normative	No improvement on the primary outcome measures (HVLT-R, WAIS-IV-digit span, verbal fluency, PCAT, simple reaction time/CRT, and the CAFS) or the additional assessments, including MMSE and the MDRS-2. Like the findings in the previous study in PDD, the overall NPI score improved due to the improvement of the neuropsychiatric symptoms. On stimulation, there was a decrease in connectivity between the posterior cingulate cortex of the DMN and the right inferior parietal lobule and an increase in connectivity between the left intraparietal sulcus and the left inferior frontal gyrus, and the left superior parietal lobule (precuneus), and the right paracingulate gyrus were noted in the FPN.
12	Oswal 2021	Prospective, based on Gratwicke 2018 and 2020	NBM	6 PDD and 5 LBD	See Gratwicke 2018 and 2020	MRI/structural and functional connectivity/normative MEG	Different networks related to NBM functional and structury connectivity. 1. A beta band network to supplementary motor area, which drives the activity in the NBM; 2. A delta/theta band network to medial temporal lobe structures encompassing the parahippocampal gyrus; and 3. A delta/theta band network to visual areas including lingual gyrus.
**Anorexia nervosa**							
1	Lipsman 2013	Prospective, phase I	Cg25	6	Anorexia nervosa	FDG-PET	After 9 months, 3 of the 6 patients had achieved and maintained a BMI greater than their historical baselines. DBS was associated with improvements in mood, anxiety, affective regulation, and anorexia nervosa-related obsessions and compulsions in 4 patients and with improvements in quality of life in 3 patients after 6 months of stimulation. These clinical benefits were accompanied by changes in cerebral glucose metabolism consistent with a reversal of the abnormalities seen in the anterior cingulate, insula, and parietal lobe in the disorder.
2	Zhang 2013	Prospective	NAcc	6 (4 imaging) and 12 HC	Anorexia nervosa	FDG-PET	The aim of this study was to explore the effect of NAcc-DBS in anorexia nervosa on brain metabolism. Compared to HC, patients with anorexia nervosa showed hypermetabolism in the frontal and limbic lobe, lentiform nucleus, left insula and left subcallosal gyrus, and hypometabolism in the prietal lobe. The hypermetabomilsm in the frontal lobe, hippocampus and the lentiform nuclues decreased after NAcc-DBS.
3	Lipsman 2017	Prospective, open label	Cg25	16	Anorexia nervosa	FDG-PET	At 12 months, DBS was associated with improvement of depression, anxiety, and affective regulation. Compared to bassline, at 6 and 12 months there was an increase of activity in the posterior brain regions and decrease of activity in the frontal brain regions, the basal ganglia, the thalamus and the cerebellum.

BMI = body mass index, CVLT-II = California Verbal Learning Test-II, CDR-SB = Clinical Dementia Rating Sum of Boxes, CRT = choice reaction times, HVLT-r = Hopkins Verbal Learning Test-revised (HVLT-R), MDRS-2 = Mattis Dementia Rating Scale-2, MEG = magnetoencephalogram, MDS-UPDRS = Movement Disorders Society Unified Parkinson's Disease Rating Scale, MRVRS = Modified Rush Video Rating Scale, PCAT = Posner's Covert Attention Test, sLORETA = surface low-resolution electromagnetic tomography, VLp = thalamic ventrolateral posterior, YGTSS = Yale Global Tic Severity Scale.

### Obsessive–compulsive disorder

OCD is a psychiatric disorder defined by the presence of unwanted intrusive thoughts or images (obsessions) and repetitive behaviours (compulsions) that are driven by anxiety, doubt, or an exaggerated sense of danger resulting in an OCD cycle. The estimated lifetime prevalence of OCD is approximately 2% in the general population (Ruscio *et al*., 2010). If left untreated, OCD can be very disabling, with more than 50% of patients reporting that the disorder interferes with their personal lives and work. Drug treatment includes the use of medications, such as serotonin reuptake inhibitors, other antidepressants, anxiolytics, and atypical antipsychotics, complemented by the use of psychological interventions, such as cognitive behaviour therapy (CBT) including exposure and response prevention methods (Denys, 2006; Szechtman *et al*., 2020). However, up to 10% of patients with OCD remain resistant to medication (Denys, 2006) and CBT/exposure and response prevention, which has a significant impact on patients’ lives and social functioning. In such cases, neurosurgical approaches should be considered in the treatment of the disorder (Alonso *et al*., 2015).

**Figure 1:**
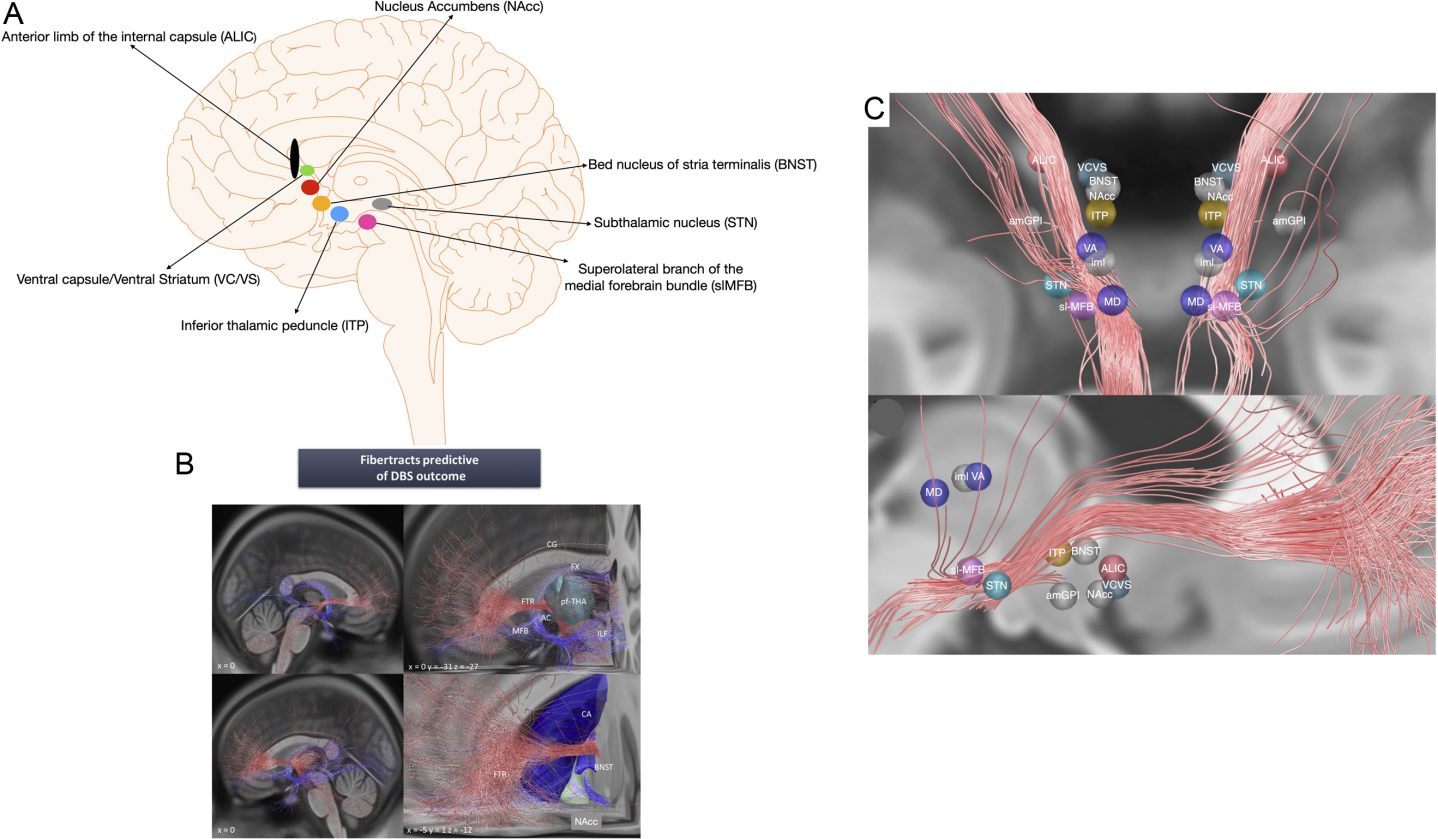
(A) The different DBS targets for OCD. (B) Fibre tracts predicting positive (red) or negative (blue) clinical outcome when associated with VTA in 22 patients with OCD treated with DBS of the ALIC/nucleus accumbens. The left panel shows a lateral view and the right panel a close-up view with labelling illustrating a strong correlation between connectivity with the anterior fronto-thalamic radiation (FTR) and clinical response. This effective fibre bundle borders the BNST and enters the ventral part of the thalamus (cyan), which is connected to the prefronta cortex (pf-THA). In addition, a negative association is evident with more ventrally located VTA connected to the slMFB or the anterior commissure (AC), whose posterior limb runs to the temporal cortex, as well as with the inferior lateral fascicle (ILF) and the fornix (FX). CA = caudate nucleus; CG = cingulum. *Reprinted and adapted with permission from Balderman*et al., *2019c*. (C) OCD targets defined in the literature in relation to the identified tract. Reported average targets mapped to standard space (upper panel: frontal representation, lower panel: sagittal representation). am-GPi = anteromedial GPi, iml = internal medullary lamina in thalamus, MD = medial dorsal thalamic nucleus, VA = ventral anterior thalamic nucleus. *Reprinted and adapted with permission from Li*et al.*, 2020*.

Neuroimaging supports the view that the pathology of OCD lies within the cortico-striato-thalamo-cortical circuity (CSTC) (Tang *et al*., 2016). Indeed, imaging has shown that patients with OCD have altered volumes, metabolism, and blood flow to the orbitofrontal cortex (OFC), cingulate, caudate, amygdala, and PFC (Tang *et al*., 2016). Neurosurgical procedures tend to target different parts of this circuity. The first modern application of DBS in psychiatry was actually in OCD (Nuttin *et al*., 1999). In this paper, the authors reported the beneficial outcome of DBS of the anterior limb of the internal capsule (ALIC) in 4 intractable cases of OCD. Since this pioneering study, different brain targets have been used to treat OCD with DBS (Fig. 1A), including the ventral capsule/ventral striatum (VC/VS) (Abelson *et al*., 2005; Greenberg *et al*., 2006; Goodman *et al*., 2010), the already mentioned ALIC (Nuttin *et al*., 1999; Anderson and Ahmed, 2003; Luyten *et al*., 2016; Barcia *et al*., 2019), and the nucleus accumbens (NAcc) (Sturm *et al*., 2003). However, it must be highlighted that these regions are anatomically related with overlap in DBS stimulation volumes, which led some to speculate that the difference between these various targets could be more semantic. Other targets involve the subthalamic nucleus (STN) (Mallet *et al*., 2002; Mallet *et al*., 2008; Chabardes *et al*., 2013), the anteriomedial part of the globus pallidus par interna (GPi) (Nair *et al*., 2014), the inferior thalamic peduncle (ITP) (Jimenez-Ponce *et al*., 2009), the superolateral branch of the medial forebrain bundle (slMFB) (Coenen *et al*., 2017), amygdala (Figee *et al*., 2013), the medial dorsal and anterior nucleus of the thalamus (Maarouf *et al*., 2016), and the bed nucleus of the stria terminalis (BNST) (Luyten *et al*., 2016). Only patients with severe or extreme OCD are considered for surgery.

An improvement of 35% on the Yale-Brown Obsessive–Compulsive Scale (Y-BOCS) has been used to assess the clinical response to DBS in OCD, and this criterion has been consistently used throughout the studies to date. OCD is the most firmly established indication for DBS in psychiatric disorders, supported by seven randomized controlled trials showing a favourable effect of DBS in different targets—VC/VS (Abelson *et al*., 2005; Goodman *et al*., 2010; Barcia *et al*., 2019), ALIC (Luyten *et al*., 2016), and STN (Mallet *et al*., 2008), BNST (Mosley *et al*., 2021), or a combination of targets (VC/VS-STN; Tyagi *et al*., 2019). Application of DBS to OCD is the only psychiatric disorder approved by the US Food and Drug Administration under humanitarian drug exemption.

The studies of DBS for OCD that used imaging reviewed here are presented in Table 1. In one of the first studies using imaging to discern the mechanism of DBS for OCD (Figee *et al*., 2013), used resting state fMRI and found that DBS of the nucleus accumbens (NAcc) (NAcc-DBS) reduced the connectivity between the NAcc, the lateral PFC, and medial PFC. Moreover, compared to healthy control participants, the connectivity was stronger in OCD patients OFF stimulation, but not ON stimulation. In addition, the DBS-induced changes in connectivity correlated strongly with the changes in obsessions and compulsions, suggesting that NAcc-DBS in OCD operates by reducing excessive fronto-striatal connectivity. Furthermore, NAcc-DBS attenuated the increase of low-frequency activity (2–5 Hz) elicited by symptoms-provoking stimuli over the frontal cortex as measured by EEG. The study showed that modulation of NAcc activity by DBS decreases the pathological fronto-striatal connectivity and reduces the low-frequency activity over the frontal cortex to a similar level as seen in healthy control participants. Similar results relating the NAcc-DBS modulation of activity of the PFC to the clinical response in treating OCD were reported in a later study (Hartmann *et al*., 2015). While the modulation of the activity of the right dorsolateral PFC (DLPFC) was associated with an excellent clinical response, modulation of the activity of the right lateral OFC and the thalamus was observed in non-responders. Furthermore, by the use of tractography in 29 healthy people, Makris *et al*. (2016) identified the connections to the lateral and medial OFC through the ventral capsule, and then compared it to a single patient operated using VC/VS-(ALIC/NAcc)-DBS who responded well to stimulation. The active contact 1 (Medtronic 3387 electrode type) was in the tracts connected to both the lateral and medial OFC, suggesting that a tractography-based patient-specific approach can enhance the efficacy of DBS for OCD. In a recent retrospective study (Liebrand *et al*., 2021), the authors analysed the pre-operative MRI in 57 OCD patients with NAcc-DBS. Voxel-based morphometry was used to investigate whether grey (NAcc) or the volume of white matter [anterior thalamic radiation (ATR)] surrounding the DBS electrode were associated with OCD severity and response to stimulation at 12 months after surgery. Machine learning was also used to predict treatment outcomes at the individual level. A larger NAcc volume before surgery was associated with lower OCD severity at 12 months, suggesting a better response to DBS in these patients, again suggesting that the effects of DBS are partly determined by individual differences in brain anatomy. However, these differences were not predictive of individual response to treatment, suggesting that structural MRI alone was not able to provide sufficient information for clinical decision making at the individual level in this study.

Further extension of these findings comes from a recent study (Baldermann *et al*., 2019c) that explored the stimulation-dependent connectivity profiles in 22 OCD patients with ALIC/NAcc-DBS. Similar to the results in previous studies (Figee *et al*., 2013; Hartmann *et al*., 2015), the degree of connectivity between stimulation the sites and the medial and lateral PFC significantly predicted clinical improvement. More specifically, there was a positive correlation between the outcome of surgery and the connectivity to the medial frontal gyrus. Even more important was the finding of a fronto-thalamic pathway, which was crucial for a beneficial outcome of the operation. This pathway goes through the ventral ALIC and connects PFC with the thalamus, passing by the NAcc, indicating that ALIC-DBS might be more effective than NAcc-DBS for treating OCD symptoms, at the same time bordering the BNST, another target used to treat OCD (Suetens *et al*., 2014; Mosley *et al*., 2021) (Fig. 1B). This indicates that a single pathway connects different targets used to treat the same condition. Moreover, connectivity of the volumes of tissue activated (VTA) with fronto-thalamic radiation was predictive of response to DBS in OCD, whereas non-response was associated with more caudally located VTA associated with the slMFB and anterior commissure. Another study by the same group (Baldermann *et al*., 2019b), looked at weight gain after VC/VS-DBS for OCD. Increase in weight after DBS, was associated with medial and dorsal localization of stimulation sites within the VC/VS and with connectivity to hypothalamic areas and the bed nucleus; once more emphasizing that localization and connectivity in DBS are not only important for predicting the overall outcome of stimulation but are also relevant for specific symptoms and side effects of stimulation.

In a small, prospective study of 7 OCD patients, Barcia *et al*. (2019), examined the overlap between projections from prefrontal areas activated during a symptom provocation task and the VTA from each electrode contact by applying probabilistic tractography to pre-operative diffusion recordings. Six patients were classified as responders, with the median symptom reduction in each patient being 50% of the best contact. This was at the caudate level in 4 cases and at the NAcc level in 2 cases. Importantly, the anatomical location of best contact (NAcc or caudate) was related to an index derived from the combination of functional MRI responses to the predominant symptom provocation and prefrontocortico-striatal projections defined by probabilistic tractography. This study was important in that it suggests that a personalized approach to targeting may lead to improved treatment outcomes in patients with OCD undergoing surgery for DBS.

Regarding slMFB-DBS, Coenen *et al*. (2017) used deterministic tractography-assisted targeting of slMFB in 2 patients. There was a positive effect of the stimulation, likely due to the modulation of ventral tegmental area activity associated with slMFB-DBS, which may also be a likely mechanism of action of STN-DBS in OCD (Voon *et al*., 2017). Liebrand *et al*. (2019) tested the hypothesis that treatment response depends on the location of active contacts in relation to ATR and slMFB in 12 patients operated on with ventral ALIC-DBS. Active stimulation of the ventral ALIC closer to the slMFB than to the ATR was associated with a better treatment outcome in these patients. Moreover, in standard space, stimulation sites were overlapping between responders and non-responders, arguing against the theoretical concept of the effect of DBS being related to a single stimulation site, and in favour of the effect of DBS being a result of modulating the brain activity at a network level. In addition, the results of this study also indicate that the effect of ventral ALIC-DBS might be actually due to the effect of stimulation of slMFB, which has already been mentioned as a separate, specific target for OCD (Coenen *et al*., 2017).

STN stimulation as a target option for OCD is due to a serendipitous discovery in a PD patient with comorbid OCD treated with STN-DBS (Fontaine *et al*., 2004). In addition to alleviation of the motor symptoms, the patient also reported a dramatic reduction in his OCD symptoms. Voon *et al*. (2017) tested 12 OCD patients with STN-DBS ON and OFF stimulation to examine the effect on reflection impulsivity on the beads task and compared the results to 24 healthy control participants. OCD patients ON stimulation showed greater impulsivity (less accumulated evidence, greater impulsive choice), but there was no difference in self-confidence ON vs. OFF stimulation. Compared to healthy control participants, OCD patients OFF stimulation accumulated more evidence. The functional connectivity based on VS and posterior putamen related to the limbic, motor, and associative parts of the STN were calculated on a set of 154 healthy people and then compared to the stimulation contacts 1 and 2 (Medtronic electrode 3389) in the OCD patients with STN-DBS. The results showed that the stimulation of the anterior associative and limb STN increases decisional impulsivity in these patients.

Stimulation of a double target, namely the anteromedial STN (amSTN) and VC/VS was an innovative approach in the study by the Queen Square group (Tyagi *et al*., 2019). Despite the small number of patients included (*N* = 6), the results of this study are important because they show that DBS significantly and equivalently reduces OCD symptoms at each site, with no additional benefit from combined stimulation. DBS of the amSTN improved cognitive flexibility, whereas VC/VS-DBS had a greater effect on mood. The VC/VS effective locus was within the VC. VC/VS-DBS was primarily associated with the medial OFC (although also with the amygdala via the amygdalofugal pathway, the habenula via the habenulointerpeduncular tract, but also with the mediodorsal thalamus and hypothalamus), and amSTN-DBS with the lateral OFC, dorsal anterior cingulate cortex (dACC), and DLPFC. Although both targets alleviated OCD symptoms, the results of this study indicate that STN and VC/VS play different roles in the pathophysiology of OCD and that the 2 DBS targets achieve their beneficial effects through different pathways.

Fridgeirsson *et al*. (2020) investigated whether the rapid effects of ventral ALIC-DBS on mood and anxiety were due to modulation of circuits primarily involving the amygdala. The improvement in mood and anxiety following DBS was associated with reduced functional connectivity between the amygdala and insula. Directional (effective) connectivity analysis revealed that DBS increased the influence of the ventromedial PFC on the amygdala and decreased the influence of the amygdala on the insula. This study highlights the role of the amygdala within the fronto-limbic network in the development of OCD symptoms, particularly when it comes to mood and anxiety. Furthermore, modulation of this network may also play a role in the clinical effects of ventral ALIC in depression.

Li *et al*. (2020) analysed data from 4 different cohorts that targeted ALIC/NAcc or the STN for treatment of OCD. A single bundle connecting the frontal lobes to the STN was identified. Anatomically, this bundle is part of the ALIC that connects PFC to the STN and the mediodorsal nucleus of the thalamus and could functionally comprise the hyperdirect prefrontal input to the STN and midbrain structures (Fig. 1C). More specifically, it connects the dorsal anterior cingulate and ventrolateral PFC to the amSTN. The main part of this bundle could represent a direct input from the frontal region to the STN. After computing the tract for either cohort alone (e.g. STN), it could be used to predict clinical improvement in the other cohort (e.g. ALIC). In addition, clinical outcomes in 2 independent test cohorts from other centres were predicted based on stimulation overlap with this tract. However, structural connectivity analysis has the disadvantage of neglecting indirect connections between whole-brain functional networks. In a recent study using the same cohorts from different centres (Li *et al*., 2021), the authors investigated whether the stimulation effects of different targets are mediated by a common or several separate functional brain networks. Results showed that optimal functional connectivity profiles had similarities and differences between target sites, while cross-predictions of improvements remained robust across cohorts and targets, suggesting a shared network. Connectivity to the ACC, insula, and precuneus was predictive of improvement regardless of stimulation target. Regions with maximal connectivity to these generally predictive areas included the insula, superior frontal gyrus, ACC, and anterior thalamus, as well as the initial stereotactic targets. This suggests that these brain regions are functionally interconnected and could be used to identify new or alternative neuromodulation targets for OCD.

In the most recent study on DBS in OCD (Mosley *et al*., 2021), the blinded phase of the study showed a significant benefit of active stimulation of BNST over sham treatment (mean difference of 4.9 points on the Y-BOCS). After the open phase, the mean reduction on the Y-BOCS was 16.6 ± 1.9 points, with 7 of the 9 participants classified as responders. CBT resulted in an additive Y-BOCS reduction of 4.8 ± 3.9 points, suggesting greater benefit from the combined therapy. An analysis of the structural connectivity of each participant's individualized stimulation field isolated right hemispheric fibres that were associated with Y-BOCS reduction. These included subcortical pathways involving the amygdala, hippocampus, and stria terminalis, as well as cortical regions in ventrolateral and ventromedial PFC, parahippocampal, parietal, and extrastriatal visual cortex. This study provided further evidence of the efficacy and tolerability of DBS in the BNST region in individuals with otherwise treatment-resistant OCD and identified a connectivity fingerprint associated with the clinical utility of the procedure. However, compared to the effect of ALIC-DBS for OCD, the reduction of Y-BOCS (Baldermann *et al*., 2019c) in this study was modest.

Several studies have used FDG-PET to study the effect of DBS in OCD. Acute high frequency DBS of VC/VS activated the OFC, the ACC, the striatum, the globus pallidus (GP), and the thalamus (Rauch *et al*., 2006), structures that together are part of the fronto-thalamo-basal ganglia-thalamic circuit implicated in OCD. Suetens *et al*. (2014) compared the surgical effects on 13 OCD patients treated with capsulotomy and 13 OCD patients operated on using BNST-DBS. The clinical effect of the 2 procedures was similar. Post-operative metabolic decreases were common in both groups especially in the ACC, PFC, and OFC. Compared to DBS, capsulotomy was associated with more pronounced and extended metabolic changes, including in the mediodorsal thalamus nucleus caudatus, cerebellum, the precuneus, the fusiform, and the lingual gyrus. Even though no connectivity analysis was done, this study indicates that two different procedures lead to similar clinical effects on the same condition. Importantly, BNST has gained an important role in the treatment of not only OCD, but also depression, anxiety, and other psychiatric disorders. This structure is also known as the extended amygdala and it is a centre of integration for limbic information and valence monitoring explaining its important, multivalent role in the treatment of psychiatric disorders (Lebow and Chen, 2016).

The acute metabolic effects of VC/VS-DBS in OCD was probed in 6 patients in an ON vs. OFF study (Dougherty *et al*., 2016). The perfusion in the dACC increased when DBS was turned ON by stimulating at the most ventral contact. This correlated with the reduction of the depressive symptoms but not with the OCD specific symptoms. When the most dorsal contact was turned ON, the perfusion in the thalamus, striatum, and GP also significantly increased. All these regions are implicated in the pathophysiology of OCD. It is important to emphasize, that in this study only the acute effect of stimulation was tested, which might explain the fact that the OCD symptoms remined unchanged, even though depression reduced when the ventral contact was turned on. In another study (Baldermann *et al*., 2019a) however, VC/VS-DBS in 3 patients significantly increased the local metabolism and lead to differential changes in various brain regions, encompassing the PFC increase of metabolism ON stimulation. Nevertheless, only 3 patients and the acute and not the chronic effect of stimulation were investigated. Change of activity in similar regions, including the basal ganglia, the cingulum, and the PFC/OFC was reported in OCD patients with ITP-DBS (Lee *et al*., 2019).

Park *et al*. (2019) compared the effects of ALIC-DBS (2 patients) and NAcc-DBS (2 patients). A greater symptom reduction was seen in patients with NAcc-DBS. The metabolism was reduced not only in the targeted limbic networks, but also in other frontal and subcortical regions. Despite the small number of patients, the results indicated that NAcc-DBS is superior to ALIC-DBS for OCD and that in addition to the local inhibition of activity of NAcc the reduction of frontal metabolism is a key mechanism of action of NAcc-DBS (Lee *et al*., 2019).

In conclusion, most studies on DBS in OCD using imaging have focused on the VC/VS, ALIC or NAcc as targets (Table 1). BNST has been used in one study (Mosley *et al*., 2021). It has been suggested that collectively these targets should be labelled as “striatal region”, different from the other targets used for DBS in OCD, such as the STN and ITP (Raviv *et al*., 2020; Li *et al*., 2021). As noted, there is now a solid imaging evidence suggesting that stimulation of either of these targets lead to similar clinical outcome as well as a reduction of the excessive fronto-striatal connectivity characteristic of OCD (Tang *et al*., 2016). Moreover, the recent studies have identified possible structural correlates, such as the fronto-thalamic pathway that connects the middle PFC with the thalamus while going through the ventral ALIC, passing by NAcc, and bordering the BNST, indicating that a single pathway actually connects different targets used to treat the same condition (Baldermann *et al*., 2019c). On the other hand, there is also evidence that stimulation of different targets has different effects on OCD symptoms. For example, DBS of the amSTN improves cognitive flexibility (greater connectivity to DLPFC and lateral OFC), whereas VC/VS-DBS has greater impact on mood (greater connectivity to the medial OFC). In general, the results of the studies reviewed here support the notion that imaging plays an important role in discerning the possible mechanism of action of DBS in ICD and might be helpful to tailor treatment for individual patient symptom profiles.

### Major depressive disorder

MDD is a common, complex, and incapacitating condition with a high prevalence worldwide of about 6% of the population and it is similar in low compared to high income countries (Kessler *et al*., 2005; Malhi and Mann, 2018; Rabin *et al*., 2020). MDD is as twice as common in women than in men (Malhi and Mann, 2018). A peak in prevalence occurs in the second and third decades of life, with a subsequent more modest peak in the fifth and sixth decades (Malhi and Mann, 2018). The pathophysiology of MDD includes dysfunction of several relevant networks in the limbic system, especially the medial PFC, amygdala, hypothalamus, periaqueductal grey, locus coeruleus, midbrain raphe, and brainstem autonomic nuclei (Vetrano *et al*., 2021). These structures are connected into a default mode network (DMN) (including medial PFC and posterior cingulate cortex), whose excessive activity relative to the task-positive network (including the associative frontal and parietal cortices) is thought to facilitate a depressive state through negative self-referential information (Price and Drevets, 2012).

DBS for MDD is reserved for patients with severe, treatment-resistant depression (TRD). Typically, these patients have tried multiple lines of therapy with no effect before being considered for DBS (Widge *et al*., 2018). The prevalence of TRD among patients with MDD is high, and according to some reports it reaches up to 30% (Rabin *et al*., 2020). MDD inclusion criteria for DBS include an age ≥30 years, a score ≥20 on the Hamilton Depression Rating Scale (HDRS), a score ≥17 on the Beck Depression Inventory scale (BDI), a duration of disease of at least 2 years, resistance to three different mechanisms of antidepressant pharmacological action, resistance to at least 6 months of psychotherapy, and resistance to electroconvulsive therapy and transcranial magnetic stimulation (Vetrano *et al*., 2021).

**Figure 2:**
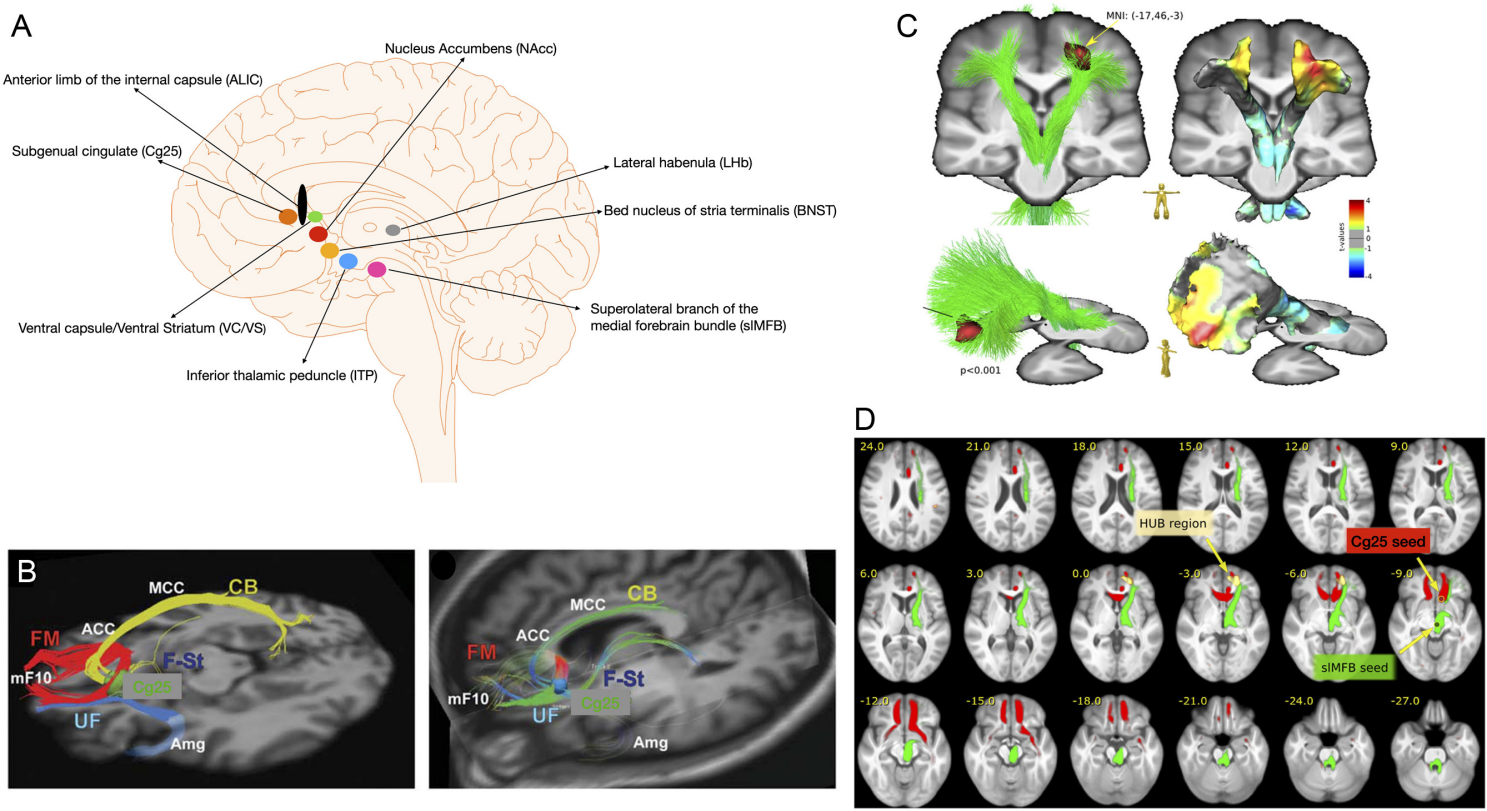
(A) The different DBS targets used for MDD. (B) Blueprint for Cg25-DBS target selection for MDD based on structural connectivity. The left panel illustrates the four necessary fibre bundles, namely the forceps minor, the fasciculus uncinatus, the cingulate bundle, and the fronto-striatal fibres. The right panel shows individualized deterministic tractography target selection in a subject: optimal target location within the Cg25 region with modelled stimulation affecting the necessary fibre bundles for effective Cg25-DBS. CB = cingulate bundle, FM = forceps minor, F-St = fronto-striatal fibres, UF = fasciculus uncinatus. *Reprinted and adapted with permission from Riva-Posse*et al.*, 2018*. (C) The fronto-orbital region associated with the slMFB was significantly enlarged in slMFB-DBS participants with MDD. In addition, enlargements of the right frontal region and symmetrical shrinkage of the posterior region were observed in this subgroup of patients. *Reprinted and adapted with permission from Coenen*et al.*, 2019b*. (D) Comparison of networks seeded by the slMFB (green) DBS target for MDD and the Cg25 (red) target from Riva-Posse 2014, Fig. 4. Both systems address the left frontal HUB region. *Reprinted and adapted with permission fromCoenen*et al.*, 2019b*.

The first study on the clinical efficacy of DBS in MDD (Mayberg *et al*., 2005) reported that 4 out of 6 patients responded to the stimulation of the subcallosal cortex (Cg25) at 6 months follow-up. A later report from the same group showed a 55% response rate at 12 months follow-up in 20 patients treated with DBS of Cg25 (Lozano *et al*., 2008). The choice of Cg25 as a DBS target for the treatment for depression was based on the previous studies (Mayberg *et al*., 1999; Mayberg *et al*., 2000) showing a persistent subgenual cingulate hypometabolism in fully recovered, previously depressed patients on long-term maintenance serotonin reuptake inhibitor treatment. The Cg25 hypoactivity was paralleled by Cg9 (PFC) reflecting the known direct reciprocal projections between these two regions (Mayberg *et al*., 1999). In addition to this target (Holtzheimer *et al*., 2017), several other targets have been used for DBS for MDD, including the VC/VS (Dougherty *et al*., 2016) and slMFB (Schlaepfer *et al*., 2013; Fenoy *et al*., 2018; Coenen *et al*., 2019a; Coenen *et al*., 2019b). Other targets include NAcc (Schlaepfer *et al*., 2008), ventral ALIC (Bergfeld *et al*., 2016), lateral habenular bundle (Schneider *et al*., 2013), and ITP (Lee *et al*., 2019). So far, five randomized controlled trials of DBS for MDD with different brain targets have been reported: VC/VS (Dougherty *et al*., 2015), vALIC (Bergfeld *et al*., 2016), slMFB (Fenoy *et al*., 2016; Fenoy *et al*., 2018; Coenen *et al*., 2019a), and Cg25 (Holtzheimer *et al*., 2017) (Fig. 2A).

The studies on DBS in MDD using imaging reviewed in this paper are presented in Table 2. In a pilot study, Schlaepfer *et al*. (2013) enrolled 7 patients with extreme TRD. They used individualized structural tractography before the operation to identify the target, slMFB, which is otherwise invisible to conventional MRI sequences. After bilateral slMFB-DBS 6 out of 7 patients were classified as responders with relatively low intensities of stimulation and a few side effects. This was the first study to show that an individualized approach in targeting using imaging might be helpful in enhancing the precision of targeting in DBS for MDD leading to clinical improvement of depression with less side effects. Moreover, the targeting of slMFB was done based on the notion that the slMFB converges towards the PFC, including Cg25 and other structures previously suggested as DBS targets for depression, such as the anterior capsule, ALIC, NAcc, and VTA (Schoene-Bake *et al*., 2010). The same approach of visualizing slMFB was used in a later study from the same group (Bewernick *et al*., 2017), in which 6 of 8 patients responded to the stimulation, four of whom went into a long-term remission.

Seventeen patients with treatment-resistant MDD underwent Cg25-DBS (Riva-Posse *et al*., 2014). After 6 months, there were seven responders, and after 2 years, there were 13. Probabilistic tractography was used to describe the white matter tracts passing through each of the activated tissue volumes, called activation volume tractography (AVT). In contrast to the non-responders, the responders all shared bilateral tracts from their activation volumes to the medial frontal cortex via the fasciculus forceps minor and the fasciculus uncinatus, to the rostral and dorsal cingulate cortex via the cingulate bundle, and to subcortical nuclei. The results of this study clearly demonstrate that patient-specific AVT modelling can identify critical pathways mediating the Cg25-DBS antidepressant response, suggesting a novel method for patient-specific selection of target and stimulation parameters.

Choi *et al*. (2015) tested the acute behavioural effects of Cg25 stimulation intra-operatively in 9 patients. Image acquisition was done before the operation. The interoceptive and exteroceptive effects of the stimulation were analysed. All 9 patients had a positive response to Cg25-DBS intra-operatively. Structural connectivity showed that the best response contacts had a pattern of connections to the bilateral ventromedial frontal cortex (via forceps minor and left uncinate fasciculus) and to the cingulate cortex (via left cingulum bundle), whereas behaviourally salient but non-best contacts had only cingulate involvement, indicating that good, and probably long(er) term effects of stimulation are due to activation of certain, depression-related networks, rather than just activation of a certain, discrete brain structure.

Even though Cg25 has been commonly used as a DBS target for MDD (Riva-Posse *et al*., 2014; Choi *et al*., 2015), results of certain studies suggest that DBS of this structure is not always successful in treating MDD. For example, in a study in which Cg25 was targeted in 5 patients (Accolla *et al*., 2016), only one responded well to stimulation 6 months after the operation. The stimulating contacts were localized in the posterior gyrus rectus bilaterally. The connectomics analysis revealed that the good effect in this “responding” patient was mediated by the modulation of circuits involving mainly the medial PFC, hence through stimulation of a different (than Cg25) node within the same cortico-subcortical circuit, indicating that this gyrus might be a good, alternative target for TRD. Similarly, in the biggest, multicentre study of Cg25-DBS for MDD (Holtzheimer *et al*., 2017) involving 90 patients, there was no difference in depressive symptom reduction between the active and sham groups.

Riva-Posse *et al*. (2018) in a prospective study of 11 patients with TRD, used deterministic tractography before Cg25 surgery so that the target met the predefined four-bundle white matter blueprint: fasciculus uncinatus, forceps minor, cingulum bundle, and fronto-striatal fibres (Fig. 2B). This four-bundle blueprint activated by Cg25-DBS was described in a previous study (Riva-Posse *et al*., 2014). The “blueprint” was used as a reference template to prospectively identify the optimal “target” on the actual deterministic tractography scan. Contacts for chronic stimulation were selected by matching the post-operative probabilistic tractography map with the pre-operative deterministic tractography map for each patient. Intra-operative behavioural responses were used as a secondary check on localization. A probabilistic tract map of all participants showed the intended inclusion of the four bundles that corresponded to the previously defined connectome blueprint. After 6 months of open stimulation, 8 of 11 patients were responders and 5 were remitters. After 1 year, 9 of 11 patients were responders, of whom 6 were in remission. One of the most important benefits of this approach was the simplified programming in the active stimulation phase during the clinical trial. This study demonstrated the use of a probabilistic tractography map for groups as a connectome blueprint for individualized, patient-specific deterministic tractography targeting, confirming previously published retrospective results (Riva-Posse *et al*., 2014).

In a single blinded trial enrolling 6 TRD patients, Fenoy *et al*. (2018) used individualized deterministic tractography and showed a positive effect of slMFB-DBS in 3 patients 1 week after the beginning of the stimulation; 1 patient dropped out, 4 of the remaining 5 had a continuing good effect of slMFB-DBS. Evaluation of modulated fibre tracts revealed significant common orbitofrontal connectivity to the target region in all responders. There was no change in the metabolism pattern of the PET scans for the patients before and after the stimulation.

Coenen *et al*. (2018) reanalysed the data of 24 slMFB-DBS patients who were implanted during two open-label trials (FORESEE and FORESEE II). We have already discussed the results of the first trial (Schlaepfer *et al*., 2013), where 6 of 7 patients were responders. The results of this study (Schlaepfer *et al*., 2013) were later replicated in a second, larger double-blind, sham-controlled study of 16 patients (Coenen *et al*., 2019a), in which all patients achieved the response criterion, 10 patients responded within 1 week, and 50% of patients were classified as remitters after 1 year of stimulation. In general, the results of studies investigating slMFB-DBS support the hypothesis of network dysfunction in TRD and the idea of DBS restoring and regulating the function of this dysfunctional network. In another analysis of the same data (Coenen *et al*., 2019b) with the goal of identifying informed features that allow prediction of treatment response to slMFB-DBS in TRD, a left fronto-polar and partly an orbitofrontal region were identified that showed increased volume in pre-operative anatomical scans (Fig. 2C). This region was designated as hitherto unknown branchpoint (HUB) region. Further statistical analysis showed that the volume of this “HUB-region” was predictive of later Montgomery–Åsberg Depression Rating Scale (MADRS) response from DBS (Fig. 2D). This study is important in that it showed for the first time a potential marker that might allow identification of responders to slMFB-DBS.

Three studies have looked at the metabolic changes related to DBS for depression (Schlaepfer *et al*., 2008; Martin-Blanco *et al*., 2015; Brown *et al*., 2020). Schlaepfer *et al*. (2008) reported the metabolic changes in the brain related to the clinical effect of NAcc-DBS in three patients with MDD. All three patients responded well on stimulation 1 week after surgery. Significant activation was noted in the DLPFC, cingulate cortex, and amygdala bilaterally. By contrast, deactivation was noted in the ventromedial and ventrolateral PFC, the dorsal caudate nucleus, and the thalamus. All these regions, components of different fronto-striatal networks, are implicated in the pathophysiology of depression.

The acute effect of Cg25-DBS on brain metabolism was tested in 7 patients with DBS (Mayberg *et al*., 2005). After 48 hours of OFF stimulation, the metabolism decreased in the dorsal anterior cingulate, premotor cortex, and the putamen, even though no acute changes in the depressive symptoms was noted. The results indicate that the DBS-related changes in the brain start before the clinical effect of stimulation becomes apparent, which is consistent with the clinical observation that at least 2 weeks delay in the emergence of a subtle worsening of symptoms after cessation of stimulation is common in DBS for depression (Mayberg *et al*., 2005). In the largest study of Cg25-DBS in depression, also using FDG-PET (Brown *et al*., 2020), 10 of 20 patients were classified as responders, 5 in the subgroup of stimulation with long pulse width, and 5 with short pulse width stimulation. Baseline Cg25 metabolism was significantly higher in responders than in non-responders, and this was predictive of a favourable clinical response of DBS. DBS decreased the activity of Cg25 6 months after the stimulation. In addition to being the largest study of Cg25-DBS using FDG-PET, the results of this study indicated that Cg25 activity as measured by FDG-PET could be used as a biomarker that can predict the outcome of Cg25-DBS that can potentially be used for prospective patient selection.

In conclusion, most of the studies reviewed here have used Cg25 as a target for DBS in patients with TRD, followed by MFB (Table 2). To the best of our knowledge, no study has probed the use of advanced imaging to assess the effect of VC/VS-DBS for MDD. In general, except for ventral ALIC (Bergfeld *et al*., 2016) the prospective, randomized controlled trails for DBS in MDD, regardless of the target—Cg25 (Holtzheimer *et al*., 2017), MFB (Fenoy *et al*., 2016; Coenen *et al*., 2019a), or VC/VS (Dougherty *et al*., 2015) have failed to meet the primary endpoints of the studies. One of the main reasons for this might be the suboptimal placement of electrodes, although other factors, such as suboptimal stimulation parameters, or suboptimal trial design might also play a role (Sullivan *et al*., 2020). The results of the studies reviewed above show that imaging can play an important role in visualizing the target structures for DBS in TRD. The strongest results supporting this idea come from the studies exploring the effect of Cg25-DBS for depression (Riva-Posse *et al*., 2014; Riva-Posse *et al*., 2018). As already mentioned, the four-bundle complex identified as important in MDD (Riva-Posse *et al*., 2014) was later prospectively used to identify the optimal target for stimulation with very good results (Riva-Posse *et al*., 2018). However, the results from these studies need to be confirmed. In addition, unlike DBS for OCD where studies directly examined the effect of stimulation at. a network level, there is a clear lack of this kind of investigation in DBS for depression. These and other issues need to be addressed in future research.

### Gilles de la Tourette syndrome

GTS is defined as a chronic neuropsychiatric disorder characterized by sudden, involuntary, repetitive, stereotyped movements or vocalizations, described as tics, typically beginning in childhood (Worbe *et al*., 2015; Pedrosa and Timmermann, 2017). GTS often presents with comorbidities such as attention deficit hyperactivity disorder, obsessive–compulsive behaviours (OCB), depression, and self-injurious behaviours. The tics are commonly preceded by a premonitory sensation and urge, which is relieved after the tics occurs. The pathophysiology of this disorder is still unclear, but some studies suggest higher cortical excitability in the primary motor cortex (M1), which may be due to underlying changes in CSTCs, similar to OCD (Leckman *et al*., 1997; Worbe *et al*., 2015). Some have proposed that GTS is a basal ganglia disorder of inhibition (Mink, 2001; Jahanshahi *et al*., 2015). More specifically, it has been proposed that tics may result from repeated inappropriate activation of striatal neurons, leading to inhibition of GPi and substantia nigra pars reticulata, the final output nuclei of the basal ganglia that would normally be tonically active to prevent unwanted movements, resulting in disinhibition of the thalamo-cortical areas to which they project (Mink, 2001). The proposal that tics may represent reduced activation of the mechanisms of habitual inhibition (Jahanshahi *et al*., 2015), has some support from an experimental study assessing habitual inhibition on a masked priming task in patients with tic disorders (Rawji *et al*., 2020). Dopaminergic hyperactivity in GTS is implicated in the generation of the tics (Leckman *et al*., 1997; Leckman, 2002). The conventional treatment of GTS involves a combination of CBT and habit reversal approaches and pharmacological therapy, mainly neuroleptics and alpha-adrenergic agonists. Even though the effect of the therapy is often satisfactory some patents are resistant to conventional treatments, at which point DBS may be considered.

**Figure 3:**
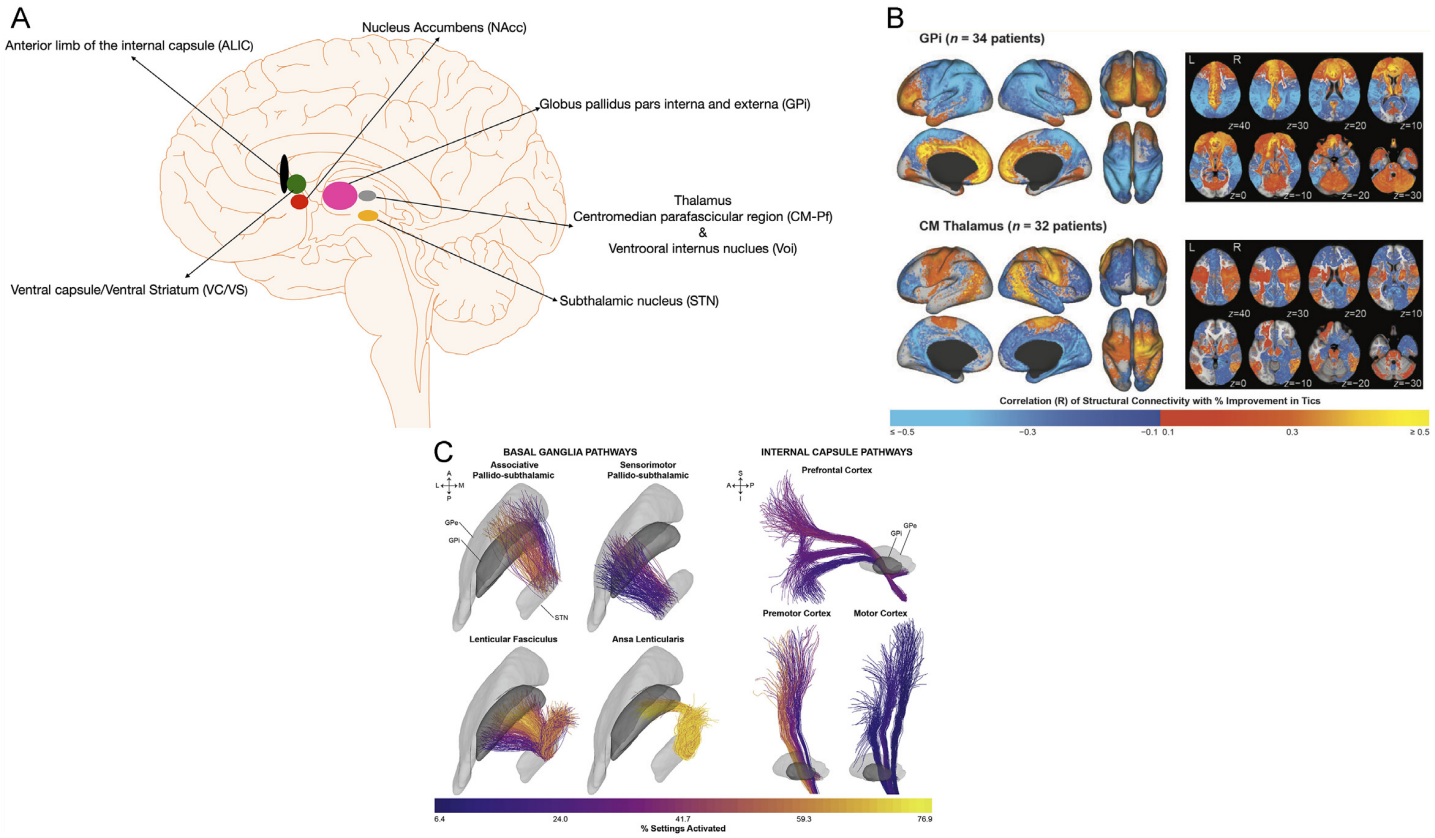
(A) The different DBS targets for GTS. (B) Stimulation-dependent connectivity to structural networks correlated with improvement in tics in patients with GTS treated with globus palidus pars interna (GPi, upper left panel) or centromedian thalamus (CM, lower left panel) DBS. The colour map refers to the correlation coefficient from the voxel-wise regression across all patients. In all patients with the GPi-DBS, connectivity to limbic networks, associative networks, the caudate, and the cerebellum was positively correlated with improvement in tics. Connectivity to sensorimotor networks and parietal-temporal-occipital networks was negatively correlated with improvement. In patients with CM-DBS, connectivity to sensorimotor networks, parietal-temporal-occipital networks, putamen, and cerebellum was positively correlated with improvement in tics. Connectivity to limbic networks and associative networks was negatively correlated with improvement. Right column, predicted improvement scores were significantly correlated with clinical improvement scores for the GPi and CM thalamus DBS. *Reprinted and adapted with permission fromJohnson*et al.*, 2020a*. (C) Activation of basal ganglia fibre tracts (left panel) and internal capsule tracts (right panel) in 35 patients with GTS after DBS of the GPi. The colour map in the panels shows the percentage of settings that activated each fibre tract in the bilateral settings across all follow-up time points for patients in the cohort. A = anterior, GPe = GP pars externa, I = inferior, L = lateral, M = medial, P = posterior, S = superior. *Reprinted and adapted with permission from Johnson*et al.*, 2020b*.

DBS for GTS was introduced by Vandewalle *et al*. (1999) stimulating the intralaminar, medial, and ventro-oral internus (VOI) nuclei of the thalamus. One DBS target used in GTS is the VOI-centromedian-parafascicular thalamic complex (VOI-CM-Pf) (Ackermans *et al*., 2011). Other targets include the GPi (Kefalopoulou *et al*., 2015), NAcc/ALIC (Kuhn *et al*., 2007), STN (Martinez-Torres *et al*., 2009), and GPe (Piedimonte *et al*., 2013) (Fig. 3A).

Studies on DBS in GTS using imaging reviewed here are summarized in Table 3. One of the first imaging studies examined the effect of DBS of CM-Pf in GTS using fMRI (Jo *et al*., 2018). The authors recorded fMRI intra-operatively during the implantation of an implanted pulse generator 1 week after the electrode implantation was done, in 5 patients under general anaesthesia. The suppression of motor and insular networks induced by thalamic stimulation correlated with the reduction of motor tics, whereas the suppression of frontal and parietal networks correlated with the reduction of vocal tics. This implies that both motor and fronto-striatal networks must be modulated if the results of the surgery are to be effective.

Brito *et al*. (2019) explored the effect of CM-Pf-DBS in 5 patients by calculating the VTA and correlating it to the change in tics on Yale Global Tic Severity Scale (YGTS). No correlation was found between the areas stimulated and the changes in tics. However, the structural connectivity estimates (using a normative connectome from healthy people) between the VTA of each patient and different regions of the brain revealed that the right frontal middle gyrus, the left frontal superior sulci region and the left cingulate sulci structurally correlated with tic improvement. This is consistent with the findings of previous studies of involvement of the PFC in the generation of tics (Moll *et al*., 1999). From a methodological point of view, the study of Brito *et al*. (2019) is important in that it shows that rather than restricting the analysis to the effect of the locus of stimulation, it is important to consider the connections of the stimulation target to other brain regions in order to show the effect of the stimulation.

In a retrospective, multicentric study, Johnson *et al*. (2019) assessed the effect of DBS over time in 110 patients operated with DBS in different targets (51 in the CM thalamus, 47 in the GPi, 4 in NAcc/ALIC and 8 in a combination of targets). The main goal of this study was to create a probabilistic stimulation atlas. In 70 patients, the contact locations were calculated and in 63 also the VTA. Tics and OCB improved over time in all groups of patients regardless of the target. The median time to reach improvement of 40% based on change in YGTS was 13 months. The active contacts were generally located near the target cores. There were regions within and surrounding the GPi and CM thalamus that enhanced tics in some patients but were ineffective in others. In addition, regions within the GPi or superior/medial to the GPi rather than inferior to the GPi were associated with greater improvement in OCB. The results of this study were limited by the fact that most of the data analysed were from open-label studies and the analysis was retrospective. Data on imaging quality were not available for all patients. However, the study is important in that it demonstrates the need to consider other types of data in addition to structural imaging in future studies of the effect of DBS in GTS.

In a later study from the same group, Johnson *et al*. (2020a) compared the effect of GPi-DBS in 34 patients and CM thalamic-DBS in 32 patients. Connectivity to limbic networks, associative networks, caudate, thalamus, and cerebellum correlated positively with improvement in tics in GPi-DBS. Regions near the anteromedial GPi had higher connectivity to positively correlated networks than the posteroventral pallidum, and VTA overlap with this map was significantly correlated with improvement in tics. In CM-thalamic-DBS, connectivity to sensorimotor networks, parietal-temporal-occipital networks, putamen, and cerebellum was positively correlated with improvement in tics. Regions in the anterior/lateral CM thalamus showed higher connectivity to the positively correlated networks but overlap of VTA with this map did not predict improvement (Fig. 3B). For OCB, both targets showed that connectivity to PFC, OFC, and cingulate cortex was positively correlated with improvement; however, only the CM thalamus maps predicted clinical outcomes across the cohort. The results of this study are of interest because they demonstrate that the structural connectivity of the stimulation site is important for symptom improvement and that the networks involved in tic improvement may differ across surgical targets. This provides an opportunity to use this approach to guide electrode placement and stimulation parameter selection, and to refine targets for GTS neuromodulation. In a further, more refined, study, the same group (Johnson *et al*., 2020b) explored the pathways with strongest association with clinical improvement in GPi-DBS for GTS. Whereas improvement in tics was associated with activation of the associative pallidosubthalamic pathway, improvement in OCB was mediated by the sensorimotor pallidosubthalamic pathway, with strong evidence for involvement of the prefrontal, motor, and premotor internal capsule pathways as well (Fig. 3C). The results of this study are surprising in that they showed that improvement in OCB symptoms was associated with the sensorimotor pathway and improvement in tics (motor phenomena) was associated with the associative pallido-thalamic pathway. This might be related to the different pathophysiology of OCB in GTS, where the repetitive compulsive behaviours are preceded by more sensory phenomena than anxiety and/or cognitive phenomena typical of OCD. This is also consistent with the findings of previous studies that metabolic changes in patients with GTS extend to motor and supplementary cortical areas in addition to limbic and associative areas.

In a prospective study, Morishita *et al*. (2020) prospectively investigated the use of CM/VC/VS-DBS in 8 patients with GTS. In general, all patients showed improvement on all measures-YGTS, Y-BOCS, and HAM-D. Using the normative connectome, the authors found that each VTA associated with therapeutic stimulation and side effects had distinctly different network properties. The therapeutic stimulation fibres were characterized by denser connections with the precentral gyrus than those of the side effects. Dizziness was associated with fibres in the cerebello-rubral network. Paraesthesia symptoms were characterized by fibres connecting the thalamus and insular cortex. Depression was characterized by fibres connecting the thalamus to the amygdala and the OFC.

Andrade *et al*. (2020) retrospectively evaluated 7 GTS patients operated using thalamic centromedian-ventro-oral internal nucleus- (CM-VOI-) DBS. They found that increasing the density of fibre projections to the seed regions of the motor cortex defined in the study—pre-SMA, SMA (supplementary motor cortex), and M1—is associated with better clinical outcomes. Activation of fibre projections to pre-SMA was higher in responders, which is consistent with findings from other studies suggesting that tic severity is associated with activation of premotor cortex (SMA and pre-SMA) (Polyanska *et al*., 2017). The non-responders showed more diffuse stimulation of multiple cortical areas simultaneously, including the pre-SMA, SMA, and M1, to which the connection was less dense compared with the responders. These results suggest that more selective connectivity leads to better clinical outcomes. Furthermore, these results and those from other studies (Kakusa *et al*., 2020) again indicate that abnormal cortical connectivity of thalamo-cortical pathways underlies GTS and that modulation of these circuits by DBS may reduce tics.

Haense *et al*. (2016) used single photon emission computed tomography (SPECT) to study the effect of GPi/CM-VOI-DBS in GTS. Pre-operatively, the perfusion was reduced in frontal, central, and parietal regions, and increased in the cerebellum. Both, the stimulation of GPi and CM-VOI increased the perfusion of the frontal region and decreased the perfusion in the cerebellum.

In conclusion, like the other conditions reviewed here, different targets have been used to treat GTS by DBS. There is currently no reliable biomarker that could be used to predict the clinical response of DBS in GTS. However, the results from the recent imaging studies reviewed here (Table 3) suggest that both functional and structural connectivity can be successfully used to investigate the effect of DBS in GTS with the goal of identifying diagnostic or prognostic markers for GTS. This is an emergent field that holds promise.

### Dementia

Dementia refers to a group of brain disorders that affect all aspects of cognitive function including attention, memory, and learning, executive functions, language, perceptual motor function, and social cognition. DSM-V criteria for diagnosis of dementia includes significant decline in one or more of these cognitive domains that interferes with a person’s social and occupational abilities and independence in daily activities. Dementia is primarily a disorder of old age and with improved life expectancy, particularly in the Western world, the prevalence of dementia has also increased. It is estimated that globally 50 million people have dementia and this could increase by 10 million new cases every year (WHO, 2020). In addition to the personal psychological, social, physical, and economic impact of dementia for sufferers, the burden of dementia extends to carers and families. Dementia is commonly treated with acetylcholinesterase inhibitors and *N*-methyl-D-aspartate–receptor antagonists, which at best produce moderate improvement of symptoms. So far, there is no cure or effective medical treatment for dementia available. The most common neurodegenerative types of dementia are Alzheimer's disease (AD), accounting for 60–70% of cases, and LBD/Parkinson's disease with Dementia (PDD) and these have been the focus of DBS treatment.

**Figure 4:**
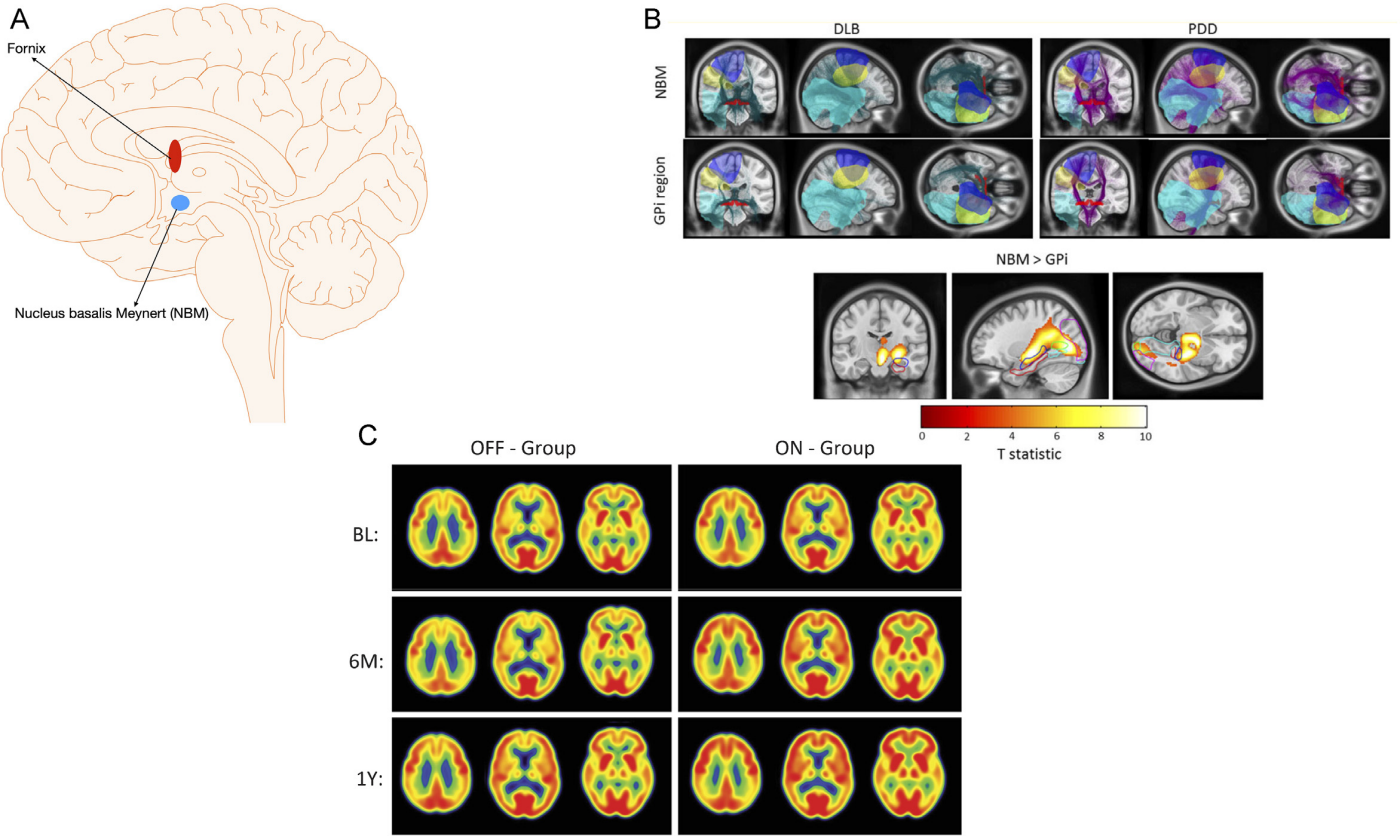
(A) The different DBS targets for dementia. (B) Top, streamlines of fibres leading to cortical regions from near the NBM and GP are shown on a T1-weighted MRI scan for the two disease groups—dementia with Lewy bodies (DLB) and PDD. In DLB patients, the fibres are coloured green, whereas in PDD patients they are magenta. Magnetoencephalogram-derived cortical networks show coherence with both GPi and NBM in the delta/theta (turquoise area). Low beta (blue area) and high beta (yellow area) bands are also shown. Bottom: a statistical parametric map shows the results of 2 × 2 ANOVA with factors “disease” (PDD versus DLB) and “contact location” (NBM versus GPi). The statistical T-scan shows regions that have significantly greater structural connectivity with the NBM than with the GPi (main effect of location). These include the hippocampus (blue contour), the lingual gyrus (turquoise contour), the calcarine cortex (green contour), and the occipital cortex (magenta contour). *Reprinted and adapted with permission fromOswal*et al.*, 2021*. (C) PET images of cerebral glucose metabolism by treatment group. Summed Axial Images of standardized update values (SUVs). Baseline (BL), 6 or 12 months after continuous bilateral DBS of the fornix for AD. Representative axial slices show that cortical glucose metabolism was stable or decreased over time in patients in the OFF group. Patients in the ON group experienced an increase in brain metabolism at 6 months, particularly in the temporal and parietal regions, which persisted at 12 months. The colour scale indicates SUVs, with red indicating the highest, yellow and green the middle, and blue the lowest. Patients took the same medications during DBS treatment from baseline to 12 months. *Reprinted and adapted with permission from Lozano*et al.*, 2016*.

The modern era of DBS for dementia was started by the group of Andreas Lozano in Toronto by a fortuitous observation during DBS in a patient treated for obesity. The patient, operated with bilateral DBS of the ventromedial hypothalamus, failed to lose weight, but showed vivid memory recollections during stimulation, which lead to the studies of what is now known as fornix DBS for AD (Hamani *et al*., 2008). However, the first report of DBS in dementia was in in the mid-1980s (Turnbull *et al*., 1985). In this case study, a 72-year-old woman with AD was operated in the left nucleus basalis of Meynert (NBM) and 50 Hz stimulation was delivered. Even though there was no clinical improvement of the cognitive decline, the temporal and parietal regions on the side of the stimulation had a preserved brain metabolism, compared to the rest of the brain, suggesting a possible role of the stimulation in neuromodulation. One of the possible reasons for the inefficacy of the stimulation was the fact the DBS was unilateral. Ever since, DBS of either the fornix or NBM has been tried in several trials to treat mostly AD (Laxton *et al*., 2010; Smith *et al*., 2012; Kuhn *et al*., 2015a; Kuhn *et al*., 2015b; Lozano *et al*., 2016; Baldermann *et al*., 2018), but also PDD (Freund *et al*., 2009; Gratwicke *et al*., 2018), and recently also LBD (Gratwicke *et al*., 2020) (Fig. 4A).

In the first case study of the effect of NBM-DBS in PDD (Freund *et al*., 2009) both the STN and NBM were targeted bilaterally in a single operation. The patient was observed for 13 months. The cognitive decline was so severe before the operation that the patient could not complete many of the planned cognitive tasks before surgery. Turning on the STN electrodes improved motor symptoms but left cognitive performance almost unchanged. Turning on NBM electrical stimulation significantly improved cognitive function in this patient. Importantly, NBM-DBS used low-frequency stimulation at 20 Hz. The same target and stimulation parameters were later used in a double-blind crossover sham-controlled study by our group (Gratwicke *et al*., 2018). However, the electrodes were positioned such that some of the contacts were in the GPi, so that in case of inefficiency of the NBM stimulation for cognitive symptoms, the GPi would be used to treat the motor fluctuations and symptoms typical of advanced PD. There was no improvement on the primary, cognitive outcome measures. However, at a group level the Neuropsychiatric Inventory (NPI) total score improved, mainly due to the improvement of visual hallucinations that are a hallmark of PDD. Interestingly, the NBM-DBS improved Movement Disorders Society Unified Parkinson's Disease Rating Scale-IV measure of dyskinesias probably due to current spread to the GPi. There was no effect of stimulation on the DMN at the group level. The same study design, with the same target nucleus (NBM) was probed in 6 patients with LBD (Gratwicke *et al*., 2020). Again, there was no significant change in any of the cognitive measures. Similar to the findings in the previous study in PDD, the overall NPI score improved due to the improvement of the neuropsychiatric symptoms. Unlike the study in PDD, there was a statistically significant decrease in connectivity between the posterior cingulate cortex of the DMN and the right inferior parietal lobule associated with active stimulation. At the same time, there were stimulation-associated increases in functional connectivity between the left intraparietal sulcus and the left inferior frontal gyrus, and the left superior parietal lobule (precuneus), and the right paracingulate gyrus were noted in the frontoparietal network. In conclusion, based on the clinical results of these studies, the stimulation of NBM in PDD and LBD did not improve cognition. However, in both groups of patients the neuropsychiatric symptoms improved. In patients with LBD, a change in the activity of the DMN and FPN was noted suggesting a possible effect of NBM-DBS on the networks responsible for modulation of attention. In the most recent study from this group, the authors leveraged the data from magnetoencephalography with the data of MRI tractography and found different networks related to the NBM functional and structural connectivity (Oswal *et al*., 2021): 1). A beta-band network to the SMA driving activity in the NBM; 2). A delta/theta-band network to the medial temporal lobe structures comprising the parahippocampal gyrus; and, 3). A delta/theta-band network to visual areas including the lingual gyrus. These functional networks of the NBM likely play important roles in motor control, memory, and visual function, respectively (Fig.   4B).

We will now focus on the studies exploring the effect of DBS for AD. Laxton *et al*. (2010) explored the effect of 20 Hz fornix DBS in 6 patients with AD in a prospective trial. DBS increased neuronal activity in the memory circuit, including entorhinal and hippocampal areas, and activated the brain DMN. PET scans showed an early and striking reversal of impaired glucose use in the temporal and parietal lobes, which was maintained after 12 months of continuous stimulation. There was no clear clinical impact of DBS on cognition, but post hoc evaluation of the cognitive subscale of the Alzheimer's Disease Assessment Scale and the MMSE in some patients suggested possible improvements and/or slowing of cognitive decline at 6 and 12 months. There were no serious adverse events. Further connectivity analysis of the data from this study (Smith *et al*., 2012) found that 1 year of DBS increased cerebral glucose metabolism in two orthogonal networks: a frontal-temporal-parietal-striatal-thalamic network and a frontal-temporal-parietal-occipital-hippocampal network. In similar cortical regions, higher baseline metabolic rate before DBS and higher metabolic rate after 1 year of DBS correlated with better outcomes in global cognition, memory, and quality of life. These studies (Laxton *et al*., 2010; Smith *et al*., 2012) served as a basis for a later study from the same group (Lozano *et al*., 2016). In this multicentre, randomized, double-blind prospective study, 42 patients with AD were treated with fornix DBS. DBS for AD was safe and associated with increased cerebral glucose metabolism (precentral gyrus, postcentral gyrus, temporal association cortex, hippocampus, parietal association cortex, occipital cortex (cuneus), and cerebellar hemispheres) at 6 months but not at 12 months post-operatively (Fig. 4C). There were no differences in cognitive outcomes for the group as a whole. However, participants aged 65 years or older may have derived some benefit from fornix DBS, whereas there may have been deterioration in patients younger than 65 years. This could be due to younger patients having worse brain pathology than older patients. Another explanation could be that the younger patients may have had different pathology than the typical AD. Fontaine *et al*. (2013) reported similar results in a single patient operated using fornix DBS in whom, after 1 year of stimulation, the memory scores [MMSE, (Alzheimer's Disease Assessment Scale-Cognitive subscale (ADAS-Cog), Free and Cued Selective Reminding Test (FCSRT)] were stabilized compared to baseline, and metabolism in the mesial temporal lobes increased bilaterally as assessed by FDG-PET.

The idea of utilizing NBM as a target for DBS in AD, first published by Turnbull *et al*. (1985) was further explored by Kuhn et al. (2015a). Based on stable or improved primary outcome parameters 12 months after surgery, 4 of the 6 patients from this phase I feasibility study were considered responders. No serious or non-communicable side effects associated with stimulation were observed. Whole-brain, parietal, and temporal metabolism, including the amygdalo-hippocampal region, increased. The mechanism of action behind the effect of NBM-DBS for AD could be related to either increased release of acetylcholine or activation of memory networks including the hippocampus. Another possibility would be activation of plasticity-related neurotrophic factors that slow disease progression. Two other younger and less affected AD patients (Kuhn *et al*., 2015b) were later operated on using the same procedure (Kuhn *et al*., 2015a). Patient 1 was stable in the first year and showed worsening ADAS-Cog 2 years later. Patient 2 was stable (ADAS-Cog), and the MMSE actually improved 2 years after surgery. The results indicate that NBM-DBS performed at an earlier stage of AD and at a younger age may have a beneficial effect on disease progression and cognitive function. A study from the same group (Baldermann *et al*., 2018), involving 6 patients from the phase I study mentioned earlier (Kuhn *et al*., 2015a), found that a fronto-parieto-temporal pattern of cortical thickness was associated with a favourable outcome. Modulation of streamlines associated with left parietal and opercular cortices was associated with better response to NBM-DBS. The results indicate that patients with less advanced atrophy may benefit from NBM-DBS and that the beneficial effects of the intervention are related to preserved fronto-parieto-temporal interaction.

In conclusion, the clinical efficacy of DBS for dementia remains to be proven. From an imaging perspective, most of the studies to date have used the nuclear-medicine (FDG-PET) imaging modalities. This is probably because FDG-PET is now an established diagnostic tool for dementia. In addition, until recently, MRI has been restricted for use in DBS before surgery only. The era of connectomic research in DBS has just started and, similar to the other indications, might prove useful not only to study dementia, but also to better target the existing or novel targets for DBS for this disorder.

### Anorexia nervosa

Anorexia nervosa is an eating disorder characterized by self-imposed drastic weight loss (or lack of adequate weight gain in growing children) (Wu *et al*., 2013). It can also be manifested by difficulty maintaining a weight appropriate for height, age, and stature, and for many sufferers, a distorted body image. People with anorexia usually restrict the number of calories and the types of food they eat. Some sufferers also exercise compulsively and use laxatives to control their weight (Wu *et al*., 2013). Serious medical morbidity and psychiatric comorbidity are almost always present, including MDD (most common) and OCD (Zipfel *et al*., 2015). In fact, anorexia nervosa can be considered an OCD that focuses on excessive control of food intake (Zipfel *et al*., 2015). As shown in genome-wide association studies (Yilmaz *et al*., 2020) anorexia nervosa is genetically very closely related to OCD. The lifetime prevalence of anorexia nervosa has been reported to be approximately 1% in women and less than 0.5% in men (Zipfel *et al*., 2015). The disease usually has a protracted and relapsing course, and the level of disability and mortality, especially without treatment, is very high. In fact, anorexia nervosa has the highest mortality rate among all psychiatric disorders and can reach up to 5.1 deaths per 1000 person-years in study populations (Zipfel *et al*., 2015).

**Figure 5:**
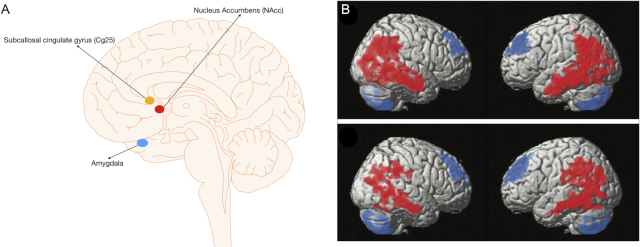
(A) The different DBS targets used to treat anorexia nervosa (Cg25, NAcc), addiction (NAcc), PTSD (amygdala), and schizophrenia (Cg25, NAcc). (B) Cerebral glucose metabolism (CGM) from 16 patients with anorexia nervosa, who had undergone DBS of the subgenual cingulate cortex. 6 months (upper panel) and 12 months (lower panel) post-operation. Blue areas show decreased and red areas show increased CGM compared to baseline. *Reprinted and adapted with permission from Lipsman*et al.*, 2017*.

Several open-label studies and a few case reports reported efficient treatment of anorexia nervosa with DBS. Among the first studies, Israel *et al*. (2010) reported a good effect of subgenual cingulum DBS to treat refractory depression and concomitant anorexia nervosa in a 17-year-old girl. Later, Blomstedt *et al*. (2017) reported a similar case of refractory depression with anorexia nervosa successfully treated with DBS of the stria terminalis. In this patient, there was a slow, but profound effect of DBS on both the depression and anorexia nervosa. McLaughlin *et al*. (2013) reported a favourable outcome of VC/CS-DBS in an OCD patient with concomitant anorexia nervosa. A good effect of different DBS targets in anorexia nervosa has been reported in several other studies: NAcc (Wang *et al*., 2013; Wu *et al*., 2013; Liu *et al*., 2020) and Cg25 and NAcc (Villalba Martinez *et al*., 2020) (Fig. 5A).

In 3 studies (Table 3), the authors used imaging techniques to detect the effect of DBS in anorexia nervosa (Lipsman *et al*., 2013; Zhang *et al*., 2013; Lipsman *et al*., 2017). In a study by Lipsman *et al*. (2013), 3 of the 6 patients had achieved and maintained a body mass index above their historical baseline at 9 months. DBS was associated with improvements in mood, anxiety, affective regulation, and compulsions and obsessions associated with anorexia nervosa in 4 patients, and with improvements in quality of life after 6 months of stimulation in 3 patients. Compared with baseline and 6 months after surgery, these clinical benefits were associated with changes in cerebral glucose metabolism that were associated with reversal of abnormalities observed in the disorder in the anterior cingulate, insula, and parietal lobes. Zhang *et al*. (2013) specifically sought to investigate the effects of NAcc-DBS at anorexia nervosa on brain metabolism. Compared with 12 healthy control participants, 6 patients from anorexia nervosa showed hypermetabolism in the frontal and limbus, nucleus lentiformis, left insula, and left subcallosal gyrus, and hypometabolism in the parietal lobe. Four of 6 patients were treated with NAcc-DBS. Hypermetabolism in the frontal lobe, hippocampus, and nucleus lentiformis decreased in these patients after NAcc-DBS. Lipsman *et al*. (2017) extended the results of the previous phase I study (Lipsman *et al*., 2013) to 16 additional patients with anorexia nervosa treated with Cg25-DBS. After 12 months, DBS was associated with improvement in depression, anxiety, and affective regulation. Compared with baseline, at 6 and 12 months there was an increase in activity in posterior brain regions (involved in body perception) and a decrease in activity in frontal brain regions, basal ganglia, thalamus, and cerebellum (Fig. 5B).

In conclusion, DBS for anorexia nervosa is still mainly an experimental procedure. The right target for treating specific comorbidities associated with anorexia nervosa and the mechanism of action are still not clear. In addition to the lack of imaging studies, there is a lack of controlled clinical studies on the efficacy of DBS in anorexia nervosa.

### Addiction, PTSD, and schizophrenia

NAcc is the main target for DBS in substance abuse and addiction because it is thought to play a role in the neurobiological circuits for craving and withdrawal. Although the mechanism of DBS in addiction is not fully understood, DBS of the NAcc is thought to modulate the mesolimbic dopaminergic system implicated in the reward circuity (Hassan *et al*., 2020; Navarro *et al*., 2021). Chronic substance abuse increases mesolimbic activity and leads to downregulation of dopamine receptors, resulting in substance tolerance and dependence. Available data indicate a beneficial effect of DBS for substance addiction (for recent reviews, please see Hassan *et al*., 2020 and Navarro *et al*., 2021). To the best of our knowledge, no study has used imaging to study the effect of DBS in substance addiction.

Only 2 cases with PTSDs (PTSD) treated with DBS of the basolateral amygdala have been published so far (Langevin *et al*., 2016). The effect of stimulation was reported to be favourable, but it is very hard to draw conclusions based on such a small number of cases (Koek *et al*., 2019).

Invasive surgical treatment options were first available for schizophrenia. Edgar Moniz performed the first lobotomy in 1935, followed by Freeman's discredited transorbital approach, which put an end to psychosurgery for some time. The term psychosurgery is still considered synonymous with an unethical treatment option for psychiatric patients. The first randomized trial in which patients with schizophrenia received DBS in the NAcc or in the subgenual ACC was completed in 2020 (Corripio *et al*., 2020). A total of 7 patients were treated: 2 out of 3 of patients with NAcc as target, and 2 out of 4 in the case of ACC achieved significant symptom improvement. Patients implanted with electrodes in the NAcc showed more significant and faster improvement. Finally, Wang *et al*. (2020) performed DBS of the habenula in two cases of schizophrenia and achieved efficacy in the first 6 months, although only one patient maintained outcome at 1 year. Neither of these studies used imaging.

## Discussion

Psychiatric disorders are a much larger cost to society than movement disorders (Trautmann *et al*., 2016; Yang *et al*., 2020). There is considerable interest in DBS for psychiatric disorders. The results of the studies published to date are suggestive of a positive effect and hence promising. However, only a total of several hundred patients with various psychiatric disorders have been treated with DBS during the last 20 years. Most of the studies are small, unblinded and non-randomized. There are numerous targets and no consensus regarding which targets are the best for a specific psychiatric disorder. The reasons for such a small number of patients with psychiatric disorders having had DBS surgery might be various. First and foremost, may be the cautious approach adopted because of the chequered past history of psychosurgery. Furthermore, there are no established inclusion criteria for DBS trials for psychiatric disorders, other than cases who have tried all standard medical and psychotherapeutic treatments and have been refractory and resistant to these are then considered potential candidates for DBS. This also related to the fact that the endpoints in the DBS trials for psychiatric disorders have been based on clinical rating scales, which are inherently subjective, and a checklist-based diagnostic approach that do not measure what DBS actually does (Widge *et al*., 2016). Furthermore, considering the cyclic nature of some psychiatric symptoms, the appropriate follow-up period after surgery are not known. To date, all the trials of DBS for psychiatric disorders had a limited follow-up to 2 years. It might be that DBS for psychiatric disorders needs a longer time for the effect of the stimulation to emerge and to become well established. Looking at other aspects of DBS for psychiatric disorders, one of the main problems in quantifying the effects is that the results of surgery and stimulation are not "visualized" and measured as in DBS for movement disorders. In addition, patient management can in some cases be much more complicated compared to movement disorders. For these reasons, imaging, and particularly connectivity analysis, might help objectify the DBS outcome in psychiatric disorders.

One important observation is that the targets for psychiatric disorders tend to be white matter tracts, which means that the effect of DBS in psychiatric disorders does not emanate from stimulation of a single brain region, but from stimulation of a network, i.e. a set of brain regions acting together to fulfil a certain function. A term closely related to a brain network is a connectome, which can be studied either by the use of resting-state-fMRI (rs-fMRI)—functional connectome, or by the use of diffusion MRI—structural connectome (Horn and Fox, 2020). Connectomic research in DBS is related to the connectomic research to map symptoms caused by focal brain lesions (Fox, 2018). Similar to the use of a normative connectome to test whether different lesion locations map to a single brain network, one can use a normative connectome to determine if DBS provides relief of disease-relevant symptoms (Horn and Fox, 2020). In addition, because DBS is a planned procedure, it offers the possibility to collect individualized connectome data prior to modulation (Akram *et al*., 2017; Akram *et al*., 2018). Without going into details, suffice to say that the use of both normative and individualized connectome approaches has certain advantages and limitations. For example, while individualized connectomes might be more disease/problem specific, they are hard to construct, and usually have a low signal-to-noise ratio and reproducibility. By contrast, normative connectomes are robust, publicly available, and relatively easy to use. That said, connectomic analysis has been used to establish a connection between the clinically observed improvement and DBS in OCD (Baldermann *et al*., 2019c), depression (Riva-Posse *et al*., 2014; Choi *et al*., 2015), and GTS (Johnson *et al*., 2020a). Furthermore, brain connectivity measures can help to link different DBS sites to treat the same disease to the same circuity. For example, ALIC (Baldermann *et al*., 2019c) and STN (Li *et al*., 2021) are two targets commonly used to treat OCD. By the use of structural connectivity analysis, not only a common bundle associated with the improvement across both DBS targets was identified, but also the tracts identified based on one target were predictive of clinical improvement with another target in different independent cohorts (Li *et al*., 2021). This means that regardless of the location, adding stimulation to a brain network causes not only local, but also long-distance changes (Sullivan *et al*., 2020). Both structures (ALIC and STN) belong to the CSTC, whose impairment is important for the development of OCD (Radulescu *et al*., 2017; Robbins *et al*., 2019). Similarly, different DBS targets used in the treatment of depression are part of a single anatomically connected circuit (Dougherty *et al*., 2015; Dougherty *et al*., 2016; Coenen *et al*., 2019a; Coenen *et al*., 2019b). However, stimulation of different targets, in addition to producing the same overall effect, might also result in different effects. For example, while the stimulation of the STN or VC/VS led to reduction of OCD symptoms, stimulation of the STN preferentially led to improvement of cognitive flexibility and ALIC stimulation resulted in improvement of the comorbid depressive symptoms (Tyagi *et al*., 2019). Therefore, the effect of stimulation of different networks are symptom specific. That is, if a network mediates a specific cognitive or affective function, whose impairment leads to a specific symptom of a psychiatric disorder, then stimulation of a specific target related to that network modulates the cognitive or affective function related to this network, and hence also the symptom of the psychiatric disorder (Horn and Fox, 2020; Sullivan *et al*., 2020). In other words, DBS in psychiatric disorders can be viewed as modulating cognitive or affective functions such as decision making, emotion regulation, and adaptation. Such an analysis has already been performed for STN-DBS for PD, such that structural connections between the STN-DBS site and the SMA were associated with an improvement in bradykinesia and rigidity, while structural connections to the M1 were associated with an improvement in tremor (Akram *et al*., 2017). Connectivity to M1 is also associated with improvement in tremor in Vim-DBS in patients with essential tremor (Akram *et al*., 2018; Al-Fatly *et al*., 2019).

Side effects emerge frequently after DBS. Imaging studies can also be used to understand the mechanism of side effects. For example, weight change after ALIC-DBS for OCD has been related to the stimulation of BNST (Baldermann *et al*., 2019b). A case report of a patient operated on using Cg25-DBS for anorexia nervosa with seizures as a side effect at suprathreshold stimulation was attributed to connectivity of the stimulation site to the hippocampi and cingulate gyri (Boutet *et al*., 2019). Therefore, imaging can be used to avoid stimulating the connections associated with side effects (Horn and Fox, 2020). DBS targeting for different indications has long been mainly based on imaging (Foltynie *et al*., 2011). Structural connectivity can add on this in identifying the precise location for stimulation, such as MFB (Schlaepfer *et al*., 2013), the intersection of the forceps minor, the cingulum and the uncinate fasciculus (Riva-Posse *et al*., 2014; Choi *et al*., 2015; Riva-Posse *et al*., 2018). This, however, needs to be considered with caution, as a precise anatomical localization of the target based on in depth understanding of the possible effects of stimulation is still of paramount importance to properly guide DBS surgery.

Most of the studies applying an imaging modality other than MRI, mainly FDG-PET, come from dementia and anorexia nervosa research (Table 3). Even though MRI-based imaging has mainly replaced the use of FDG-PET, we believe that there is still a place to use PET studies in DBS research, as they provide different types of information. Namely, while PET provides information on the focus of abnormalities (e.g. hypometabolism and reduced target binding), fMRI is used to analyse functional connectivity (Watabe and Hatazawa, 2019). This area, particularly the combined use of PET and fMRI-based connectivity analysis, is not well understood and needs further exploration.

In conclusion, imaging, and especially connectivity analysis, offers exceptional opportunities to better understand and even predict the clinical outcomes of DBS. This especially hold true for DBS for psychiatric disorders in which there is a lack of objective biomarkers needed to properly guide DBS pre- and post-operatively. In future, imaging might also prove useful to individualize DBS treatment. Last, one of the most important aspects of imaging in DBS is that it allows us to better understand the brain through observing the changes of the functional connectome under neuromodulation.
